# Supplementary material for: Multilineage Differentiation for Formation of Innervated Skeletal Muscle Fibers from Healthy and Diseased Human Pluripotent Stem Cells
Source: Cells. 2020 Jun 23;9(6):1531. doi: 10.3390/cells9061531 (PMC7349825; doi:10.3390/cells9061531)
Supplement: Supplementary file 1 [file cells-09-01531-s001.zip › cells-821883 supl final.pdf]

Materials & Methods.

Cell Lines

The C5 and H clones were derived from primary fibroblasts (Ref AG08498). The C4 clone was derived from primary fibroblasts (Ref AG04148). Fibroblasts were obtained from the Coriell Institute and previously described [1,2]. The 14,586 hiPSC clone was derived from a patient with a severe clinical FSHD carrying two normal 4q35 alleles but a heterozygote mutation in the *SMCHD1* gene (c.573A>C; p.Q193P; FSHD2, male, age 67 at sampling) causing a lack of activity of the ATPase domain [2]. hiPSCs for DMD (GM25313, male, age 13 at sampling, Ex45 del) and DM1 (GM24559, female, age 2 at sampling, CTG repeat of 1600 in the *DMPK* gene) were purchased from the Coriell Institute. Primary fibroblasts from a patient affected with limb girdle muscular dystrophy (LGMD2A, GM03932, Male, age 67 at sampling) were obtained form the Coriell Institute and reprogrammed as described above (clone LMD932C). We also derived hiPSCs for another DMD patients using fibroblasts purchased from the Coriell Institite (GM03429, male, Age 6 at sampling; Ex45-50 Del). For clones obtained in our laboratory, Individuals have provided written informed consent for the use of their sample for medical research. The study was done in accordance with the Declaration of Helsinki.

Generation and Culture of hIPSc

Human iPSCs were generated after nucleofection of primary fibroblast with episomal vectors containing *OCT3/4, SOX2, KLF4, C-MYC* and shRNA against p53. HiPSCs colonies were picked about 2 weeks after nucleofection based on ES cell-like morphology. Colonies were grown and expanded in mTeSR1 medium (Stemcells, Grenoble, France) on BD Matrigel™ (BD Biosciences, cat, No. 354277) coated dishes. HiPSCs clones were fully characterized using classical protocols as previously described [1,2].

Cell Differentiation

At day 0, clumps of cells were mechanically dissociated and transferred in a new Matrigel-coated dish and cultured in Differentiation Medium (DM; Neurobasal medium supplemented with N2 (1X) B27 (1X), P/S (1X), NEAA (1X), Glutamax (1X) (Life Technologies, Carlsbad, CA, USA)), ITS-A (1X, final concentration; life technologies, ref 51300044), LDN 193,189 (0.5 μM, Sigma, SML0559) and CHIR99021 (3 μM, Sigma Aldrich, Lyon, France, 5ml-1046). All reagents are diluted in N2. At day 6, medium is changed with a medium containing DM, LDN 193189, IGF-1 (4 ng/mL, Peprotech, No.100-11) and HGF (10 ng/mL, Peprotech, No.100-39) and β Mercaptoethanol (ThermoFisher scientifcs, Carlsbad, CA, USA, No 31350010). At day 8, HGF is removed and medium is changed with DM supplemented with IGF (4ng/mL, Peprotech, Neuilly sur Seine, France, No.100-11), β Mercaptoethanol. At day 12 medium is changed with DM supplemented with IGF (4ng/mL, Peprotech, No.100-11), β Mercaptoethanol and DAPT (10 μM, Tocris Biosciences, Bristol, United Kingdom). Medium is changed every day until Day 17 where medium is changed with DM and IGF (4ng/mL, Peprotech Neuilly sur Seine, France, No.100-11). In situations where muscle differentiation is delayed or where a poor enrichment in muscle fiber is observed, HGF and Basic Fibroblast Growth Factor (FGF2) can be added to the maintenance medium for 7 additional days.

RNA Extraction, Quality Control, Library Preparation and Sequencing

Total RNA was extracted using the RNAeasy kit (Qiagen, Hilden, Germany) following manufacturer's instructions. Quality, quantification and sizing of total RNA was evaluated using the RNA 6000 Pico assay (Agilent Technologies Ref. 5067-1513) on an Agilent 2100 Bioanalyzer system. The RNA integrity number (RIN) was calculated for each sample and only samples with a RIN >9 were kept for further use. Genomic DNA was enriched in protein-coding sequences using the in-solution exome capture SeqCap EZ MedExome Kit (Roche Daignostics, Meylan, France) according to the manufacturer's protocol. Libraries were constructed using 2 μg of total RNA after Qubit

quantification. The TruSeq Stranded mRNA Library Preparation Kit High Throughput (Illumina, San Diego, CA, USA, ref RS-122-2103) was used according to the manufacturer's guidelines. Briefly, PolyA+ containing RNA molecules were purified using polyT oligo-attached magnetic beads. Thermal fragmentation was carried out after two rounds of enrichment for PolyA+ mRNA. cDNA was synthesized using reverse transcriptase (Superscript II) and random primers. This was followed by second strand cDNA synthesis, end repair process, adenylation of 3′ ends and ligation of the adapters. The products were then purified and enriched with 15 cycles of PCR to create the cDNA library. Libraries were quantified by qPCR using the KAPA Library Quantification Kit for Illumnia Libraries (Roche, ref. 7960140001). Library profiles were assessed using the DNA High Sensitivity LabChip Kit (Agilent Technologies, Santa Clara, CA, USA Ref. 5067-4626) on an Agilent Bioanalyzer 2100. Libraries were sequenced on an Illumina Next Seq 500 NextSeq using cartridge of the NextSeq 500/550 High Output v2 kit (150 cycles) (Illumina FC-404-2002).

RNA-Seq data Processing and Differential Expression Analysis

We assessed fastq sequence data quality using FastQC v0.11.5 (Andrews, 2010) and trimmed the reads to remove adapter sequences and low-quality bases using Trimmomatic v0.36 [3]. The resulting trimmed paired-end reads were aligned using STAR v2.5.3a [4] to the GRCh37.p13 human genome release. DEGs were identified using StringTie v1.3.3 and R package edgeR v3.18.0, where raw read counts per gene were normalized using Trimmed Mean of M-values (TMM). Differentially expressed genes were extracted for a fold-change cutoff of 2 and a p value < 0.001. We used the Ensembl human gene IDs identified in the edgeR analyses as input for further analyses. Overrepresentation test analyses were performed using enrichGO from the R package clusterProfiler version 3.10.1 [5]. We identified biological processes with an FDR corrected p-value < 0.05 using an universe of 16,034 genes (39092 "genes" including variants downsized to 16,304 by enrichGO) as reference (D8, D17 and D30 genes with TPM > 0). Results are presented as dotplots with on the *x-axis* the gene count per GO term and on the *y-axis* the identified GO term. Dot size refers to the gene ratio and the color scale represents the adjusted p-value.

Heatmaps were obtained thanks to the pheatmap version 1.0.12 R package using TPM values (https://www.rdocumentation.org/packages/pheatmap). Clustered heatmaps use Ward.D2 as cluster method and Manhattan as distance metric (both on row and column). All heatmaps are scaled by row and the color scale is made from the Z-score. We used gene symbol of identified Ensembl human gene IDs obtained through DESeq2 analyses as input for further analyses.

RNA-seq data and raw count matrix were deposited at the NCBI Gene Expression Omnibus (https://www-ncbi-nlm-nih-gov.gate2.inist.fr/geo/) under the accession GSE142042 using the following token: ujsziqcmlpkbvyv.

Gene Expression Analysis and RT-qPCR.

Total RNA was extracted using Trizol (Invitrogen, Cat. No 15596-026). Reverse transcription of 1µg of total RNA was performed using the Superscript III kit and oligo dT following manufacturer's instructions at 42 °C for 50 min followed by inactivation at 70 °C for 15 min (Life Technologies). Primers were designed using Primer Blast. PCR amplification was performed on a LightCycler 480 (Roche) using the SYBR green master mix using previously described conditions [6]. Crossing-threshold (Ct) values were normalized by subtracting the geometric mean of three housekeeping genes (*GAPDH*, *PPIA* and *HPRT1*). All Ct values were corrected by their PCR efficiency, determined by 1:2 or 1:4 cDNA dilution series. Results were treated with the GraphPad prism software for statistical tests (ANOVA, Kruskal-Wallis multiple comparison test; alpha set at 0.05). Only p-values less than 0.05 were considered statistically significant.

Western Blot

Proteins were resolved on a 3-8% MOPS-NuPAGE gel (Life Technologies) and transferred on a PVDF membrane (Milipore, Burlington, MA, USA). Anti-Actin (Millipore MAB1501R) antibodies were used as loading control. Incubation with primary antibodies (MYH8 (1/1000; NOVUS NBP2-

41309), MYH 3 (1/1000; Santa cruz SC-53091), MYH2 (1/1000; Santa cruz SC-53095), MNX1 (2/1000; Millipore ABN174), DES (1/1000; Abcam, Cambridge, United Kingdom, AB15200), PAX7 (1/1000 abcam ab187339)) was done overnight at 4 °C. After 3 washes in PBS-T, anti-mouse IgG secondary antibody coupled to HRP (ThermoFisher) was incubated for 60 min (1/10 000). Proteins were revealed by chemiluminescence (ECL, Milipore, WBKLS0100) and visualized using a Quantity One Bio-rad camera. Quantified results were treated with the GraphPad prism software for statistical tests (ANOVA, Kruskal-Wallis multiple comparison test; alpha set at 0.05). Only p-values less than 0.05 were considered statistically significant.

Immunocytochemistry

Cells are fixed in 4% paraformaldehyde, washed with PBS, permeabilized and incubated in blocking buffer containing 3% BSA and 0.8% Triton for 1hour at room temperature. Incubation with primary antibodies (anti-Desmin (1/100; Abcam AB15200), anti-PAX3 (80/1000; DSHB), anti-MYH 2 (1/1000; santa cruz SC-53095), MYH 3 (1/100; santa cruz SC-53091), anti-TTN (96/100; DSHB 9D10), anti-Neurfilament M (1/2000; biolegend PCK-593P), MYH8 (1/100; Novus Biologicals NBP2-41309), anti-Dystrophin (106/1000; DSHB MANDRA 1), anti-MNX1 (1/100; Millipore ABN174), anti-MyoD (1/200; Novus Biologicals, Centennial, CO, USA NBP1-54153), anti-Islet1 (1/100; Neuromics, Edina, MN, USA GT15051)), was done overnight at 4 °C in BSA 3%, 0.8% Triton X100. After incubation, cells were washed in PBS and incubated with Alexa Fluor-non conjugated secondary antibodies (anti-Mouse Alexa Fluor 647 (1/1000; life technologies, A11034), anti-Rabbit Alexa Fluor 555 (1/1000; cell signaling, 44135) or anti-goat Alexa fluor 488 (1/1000; life technologies)) α-Bungarotoxin conjugate (1/400; ThermoFisher B35451) in the presence of 3% BSA; 0.8% Triton X100 for 1 h. Nuclei were counterstained with DAPI. Images were taken using a confocal imaging system (LSM 800, Zeiss, Oberkochen, Germany) with a 63x water-immersed lens (63x/1.20 W Korr UV VIS IR, C-Apochromat; Olympus, Shinjuku, Japan).

Imaging and Analysis of Calcium Transients

Calcium transients were measured after addition to the cell culture medium of FLUO-8AM fluorescent Ca^{2+} indicator (AAT Bioquest, Sunnyvale, CA, USA). Cells were incubated for 30min at 37°c in DM+I medium containing 5μM Fluo8-AM and 0.04% Pluronic acid before imaging on a Fast Imaging Observer system (Axio Observer.Z1/7; Zeiss) with a 10X lens (10x/0.30 M27 DIC I EC Plan-Neofluar; Olympus). Fluorescence was excited at 488nm and emission collected at >509 nm. Images were acquired as time series. Different regions were tracked to record the fluorescent intensity at a 10X magnification during 30 s and an interval of 200,0 ms corresponding to 151 frames. Analysis was performed using the Zen pro software (Zeiss). ROI was created for selected fibers then intensity mean for each fiber was exported and Calcium transients were calculated by counting the number of peaks intensity for each graph.

Cell Treatment

For drug treatment, cells were seeded and differentiated in 6 to12-well plates for 45 days. We tested the activity of Mexiletine (M2727, Sigma Aldrich, 10; 100μM diluted in methanol), Carisoprodol (C8759, Sigma Aldrich, 0.5μM; 1μM diluted in ethanol), Salbutamol (S8260, Sigma Aldrich, 1μM; 10μM, diliuted in methanol) and L-Glutamic acid potassium salt monohydrate (G1501, Sigma Aldrich, 2 μM; 20 μM diluted in H_2O) added at 0.1% in the culture medium on fiber contractions. Cell contractions were captured as described for Calcium handling measurements.

Electron Microscopy

After 5 min of wash with 0.1M sodium cacodylate buffer, cells were directly fixed in solution with glutaraldehyde 2.5% in 0.1M sodium cacodylate buffer during 1h at room temperature, then 3 times washed for 10 min with 0.1M cacodylate buffer. Cells were post-fixed with 2% osmium tetroxyde's (in 0.1M cacodylate buffer) steam during 45 min, then they were washed again 3 times

for 15 min with distilled water. Contrast with 1% Uranyl acetate in water was performed overnight at 4 °C. After 3 washes with distilled water, progressive deshydratation is requiered with 50% to 100% Ethanol baths before start the embedding in Epoxy resin (EPON 812) from 33% to 100% resin, then overnight polymerisation at 55 °C. Wells are prepared to make apico-basal axis blocks to cut. Ultrathin 60nm sections were obtained using Ultracut-E ultramicrotome (Reichert-Jung, Southbridge, MA, USA).

Pictures were obtained using JEM-1400transmission electron microscope (JEOL, Tokyo, JAPAN) at 80kV with Megaview III Camera (SIS Imaging, Münster, Germany).

Table S1. Sequence of the primers used for RT-PCR.

Gene	Forward Primer	Reverse Primer
AGRN	GTCATCAGGAGCAGGGAGC	CCTTCAGGTTGAGGACGGTG
ChAT	AGAAGCAGAAATGCAGCCCT	GCTCTCACAAAAGCCAGTGC
DES	CCGCCATCTGCGCGAGTACC	TGCTCAGGGCTGGTTTCTCGGA
GAPDH	TGC ACC ACC AAC TGC TTA GC	GGC ATG GAC TGT GGT CAT GA
MNX1	CCGGCGGATGAAATGGAAAC	GGGTCACTGTCCCTCAAGTC
ISL1	TGTTTGAATGTGCGGAGTG	GCATTTGATCCCGTACAACC
MGN	AGG AGG AGC GTG ACC TCC GC	CGT GAG CAG ATG ATC CCC TGG G
MYF6	TGCAGGAGCTGGGGGTGGAC	CTTGCTCCTCCTTCCTTAGCCGT
MYF5	AGAACTACTATAGCCTGCCGG	ATCTGTGGCATATACATTTGATACA TCA
MYH2	GGAGCTGGTGGAGGGGCCAA	TGCTCCATGGCACCAGGAGTTT
MYH3	GCTTGTGGGCGGAGGTCTGG	AGGGCTGGTTCTGAGCCTCGAT
MYH4	AGGAAAGGAGCAGCCTCCCCA	TGTCTGCTTTGAGCCTGCCACC
MYH7	GGC ACG AAG GGC TTG AAT GAG GAG	ATG GGG CTT TGC TGG CAC CT
MyH8	TCCACCAAGACCCCAGAGAGTGG	TGGGCCTCAATCCGCTCCTT
MYOD1	TGCGCAACGCCATCCGCTA	GGGCCGCTGTAGTCCATCATGC
PAX3	CACCAGGCATGGATTTTCC	TTGTCAGGAGTCCCATTACCT
PAX7	CCACAGCTTCTGCAGCTACTCTG	GGGTTGCCCAAGATGCTG
RYR1	GGCGGGAGATATACAGTCCGGTGG	AACGGGTCTTGGCCAGCGTG
SCG	TCTCCAGGGACAGTTGCTGA	TACTGCTCACGCACCATCTTAG
TTN	CCGAAATGCATCAGTCAGCG	CCTTGCAAGCTTGTGTCACC

Supplementary figures

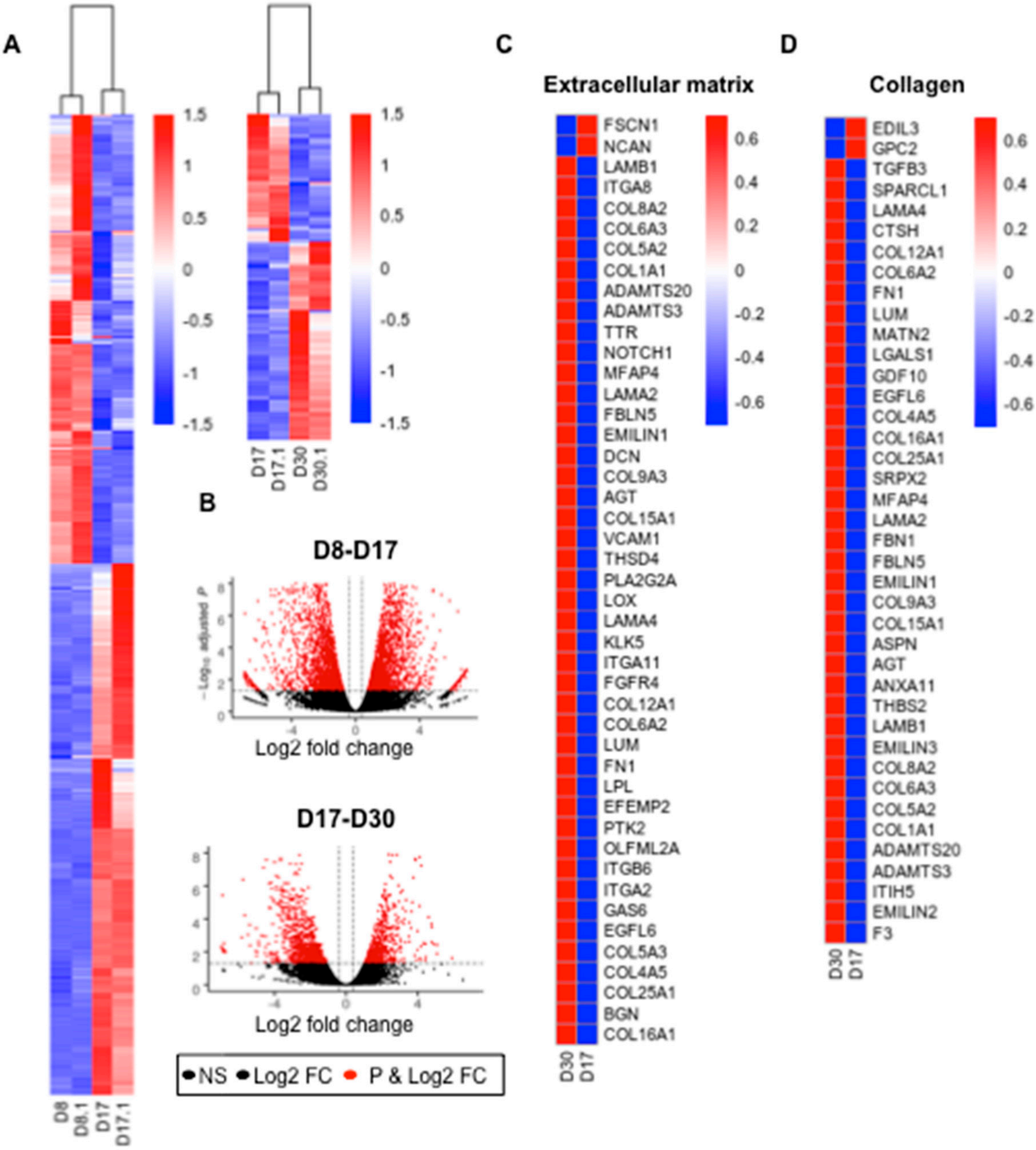

Figure S1. Expression profiling during hiPS differentiation. (**A**) Heatmap of RNAseq data (TPM values, distance: Manhattan, Clustering: Ward.D2) for gene expressed between D8 and D17 and D17 and D30 post differentiation. Unsupervised hierarchical clustering revealed two dendrogram branches that separate each time point. (**B**) Volcano plots of genes differentially expressed between D8 and D17 (upper) and D17-D30 (lower panel). (**C**) Heatmap for genes corresponding to the "extracellular matrix" GO term. (**D**) Heatmap for genes that belong to the Collagen family.

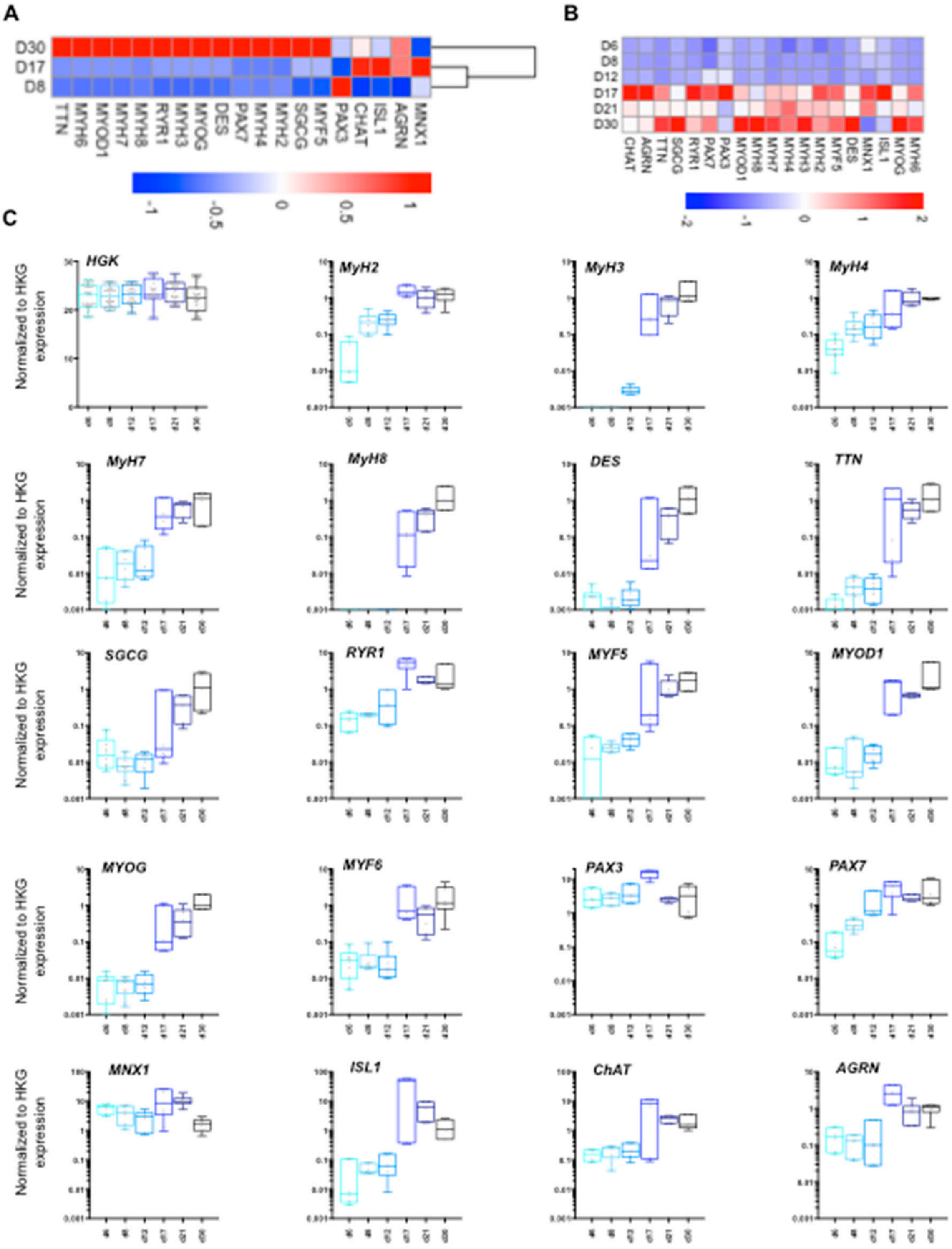

Figure S2. Time course expression of myogenic and motor neurons markers in control hiPSC-derived muscles. (**A)** Heatmap of RNAseq data (TPM values, distance: Manhattan, Clustering: Ward.D2) for gene used for validation. Consistent with the progressive increase in muscle differentiation upon DAPT addition, unsupervised hierarchical clustering revealed two major dendrogram branches that separate two different groups before and after DAPT addition at D17, maturation of skeletal muscle fibers and acquisition of functional features. (**B)** Heatmap for expression of different muscle and neuronal markers over time for control hiPSCs determined by RT-qPCR at D6, D8, D12, D17, D21 and D30. The name of the different genes is indicated on the bottom. (**C)** Expression of different muscle and neuronal markers over time for control cells at D6, D8, D12, D17, D21 and D30 post differentiation

as determined by RT-qPCR. Expression of the different genes was normalized to the expression level of three different housekeeping genes (*GAPDH*, *HPRT* and *PPIA*, first upper panel*)* and to the expression of the different genes at day 30 (ΔΔCT method, *n* = 6; biological triplicates in technical duplicates). Means ± SEM and statistical significance are reported; Kruskal Wallis multiple comparison test, α = 0.05). Expression of different Myosin Heavy Chain genes (*MyH2*, *MyH3*, *MyH4*, *MyH8*) at the different time points and expression of sarcolemmal proteins such as Desmin (*DES),* a muscle specific class III intermediate filament responsible for connecting myofibrils to each others and to the plasma membrane, *SCGG* encoding a sarcolemmal transmembrane glycoprotein that interacts with Dystrophin, *TITIN* (*TTN*) and *RYR1* (Ryanodine Receptor 1), responsible for Calcium release in the sarcoplasmic reticulum and connection to the transverse tubules, Expression of the different transcription factors required for skeletal muscle commitment and differentiation (*MRF4*, *MGN*, *MYF5*, *MYOD1*). Expression of the Paired box protein genes, *PAX3* and *PAX7*. Expression of MNX1 (HB9 homeobox gene 9), *ISLET1* (ISL LIM homeobox 1) motor neurons markers, Cholin-O-Acetyltransferase gene (*ChAT*) expressed by cholinergic neurons and AGRIN required for formation of neuromuscular junction (*AGRN).*

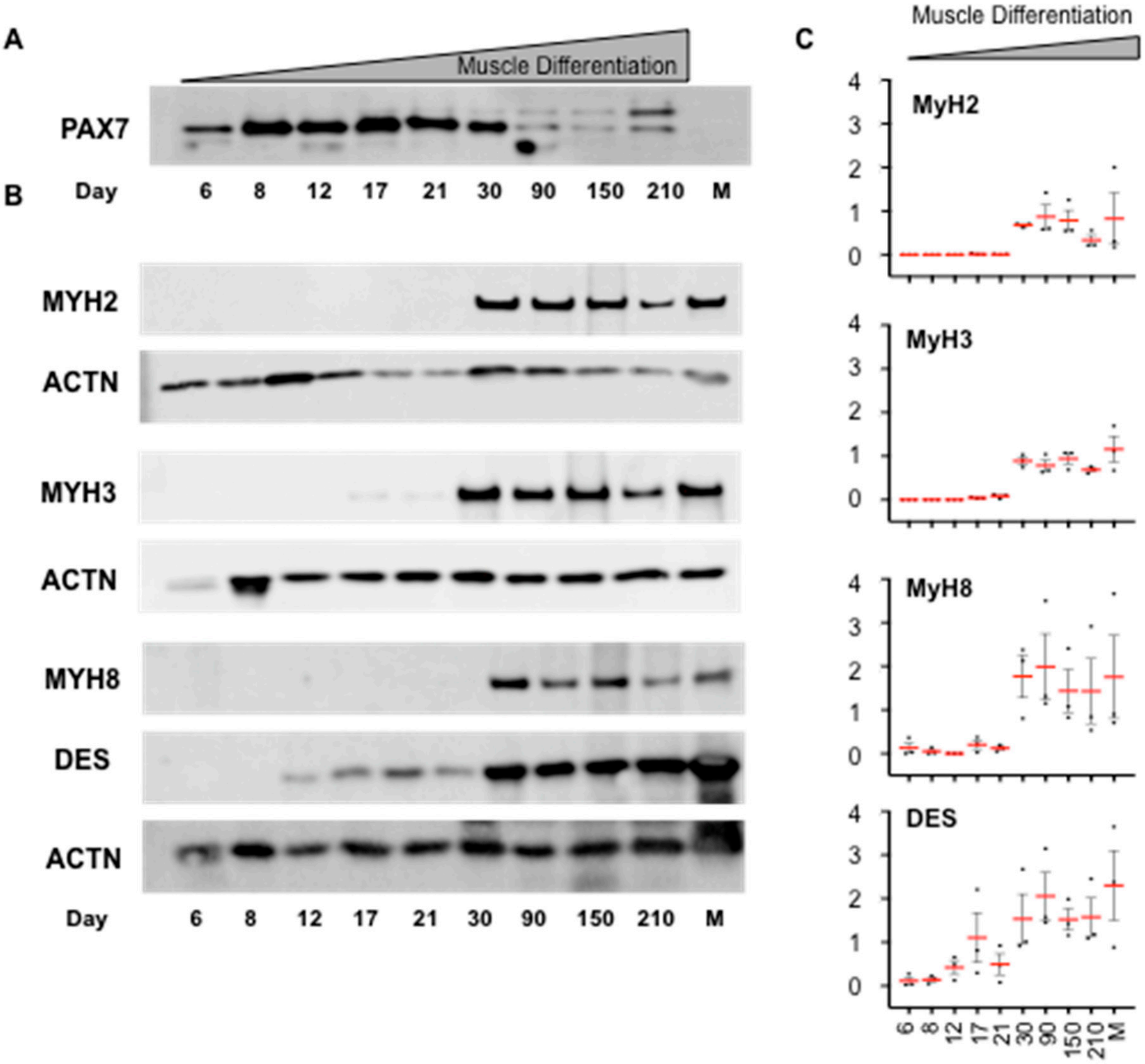

Figure S3. Expression of myogenic markers and persistence of satellite cells over time. (**A**) Western blot on whole cell extracts performed at different time points post differentiation, D6, 8, 12, 17, 21, 30 but also 90 (3 months), 150 (5 months) and 210 (7 months) days post differentiation using antibodies against PAX7. Human primary myoblasts (M) were used as control. (**B)** Western blot on whole cell extracts was performed at different time points post differentiation, D6, 8, 12, 17, 21, 30, 90 (3 months), 150 (5 months) and 210 (7 months) days post differentiation using antibodies against MyH2, MyH3, MyH8 or Desmin. Beta Actin was used as loading control. Representative western blots are presented

together with (**C)** quantification of ratio between muscle markers and Beta Actin at the different time points. Quantification corresponds to the average of three independent experiments.

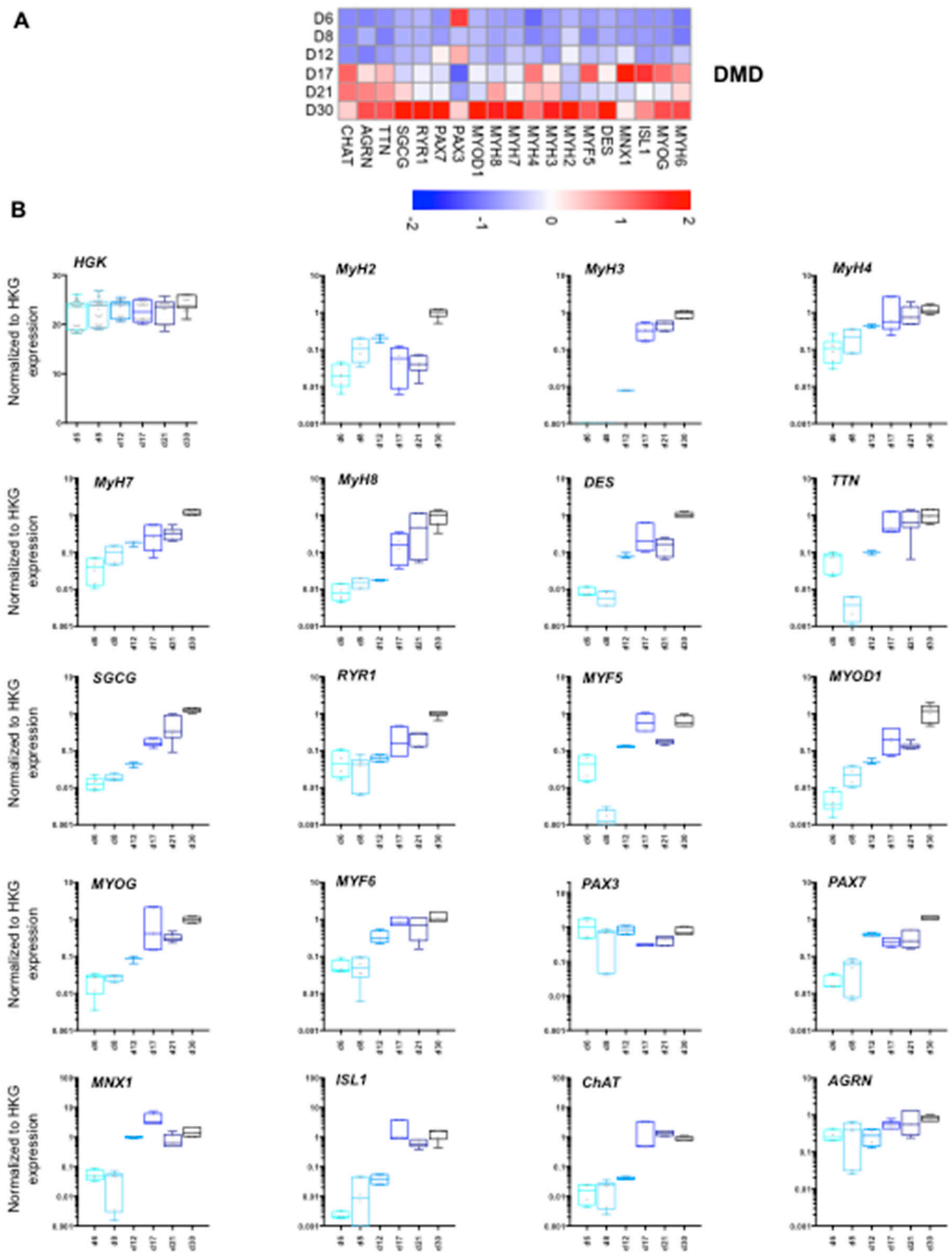

Figure S4. Time course expression of myogenic and motor neurons markers in DMD hiPSC-derived muscles. (**A**) Heatmap for expression of different muscle and neuronal markers over time for control cells at D6, D8, D12, D17, D21 and D30 post differentiation as determined by RT-qPCR for cells derived from the GM25313 hiPSC clone. The name of the different genes is indicated underneath the heatmap. Expression of the different genes was normalized to the expression level of three different housekeeping genes (*GAPDH*, *HPRT* and *PPIA)* and to the expression of the different genes at day 30 ($\Delta\Delta$CT method, n = 6; biological triplicates in technical duplicates). Means ± SEM and statistical significance are reported; Kruskal Wallis multiple comparison test, α = 0.05). (**B**) Expression of different Myosin Heavy Chain genes (*MyH2*, *MyH3*, *MyH4*, *MyH8*) at the different time points and

expression of sarcolemmal proteins such as Desmin (*DES),* a muscle specific class III intermediate filament responsible for connecting myofibrils to each others and to the plasma membrane*, SCGG* encoding a sarcolemmal transmembrane glycoprotein that interacts with Dystrophin*, TITIN* (*TTN*) and *RYR1* (Ryanodine Receptor 1), responsible for Calcium release in the sarcoplasmic reticulum and connection to the transverse tubules, Expression of the different transcription factors required for skeletal muscle commitment and differentiation (*MRF4*, *MGN*, *MYF5*, *MYOD1*). Expression of the Paired box protein genes, *PAX3* and *PAX7*. Expression of MNX1 (HB9 homeobox gene 9), *ISLET1* (ISL LIM homeobox 1) motor neurons markers, Cholin-O-Acetyltransferase gene (*ChAT*) expressed by cholinergic neurons and AGRIN required for formation of neuromuscular junction (*AGRN).* Compared to controls, expression of *MYF6* (MRF4) is detectable earlier in DMD. *MNX1* (HB9) and *ChAT* expression is delayed while AGRN is activated earlier.

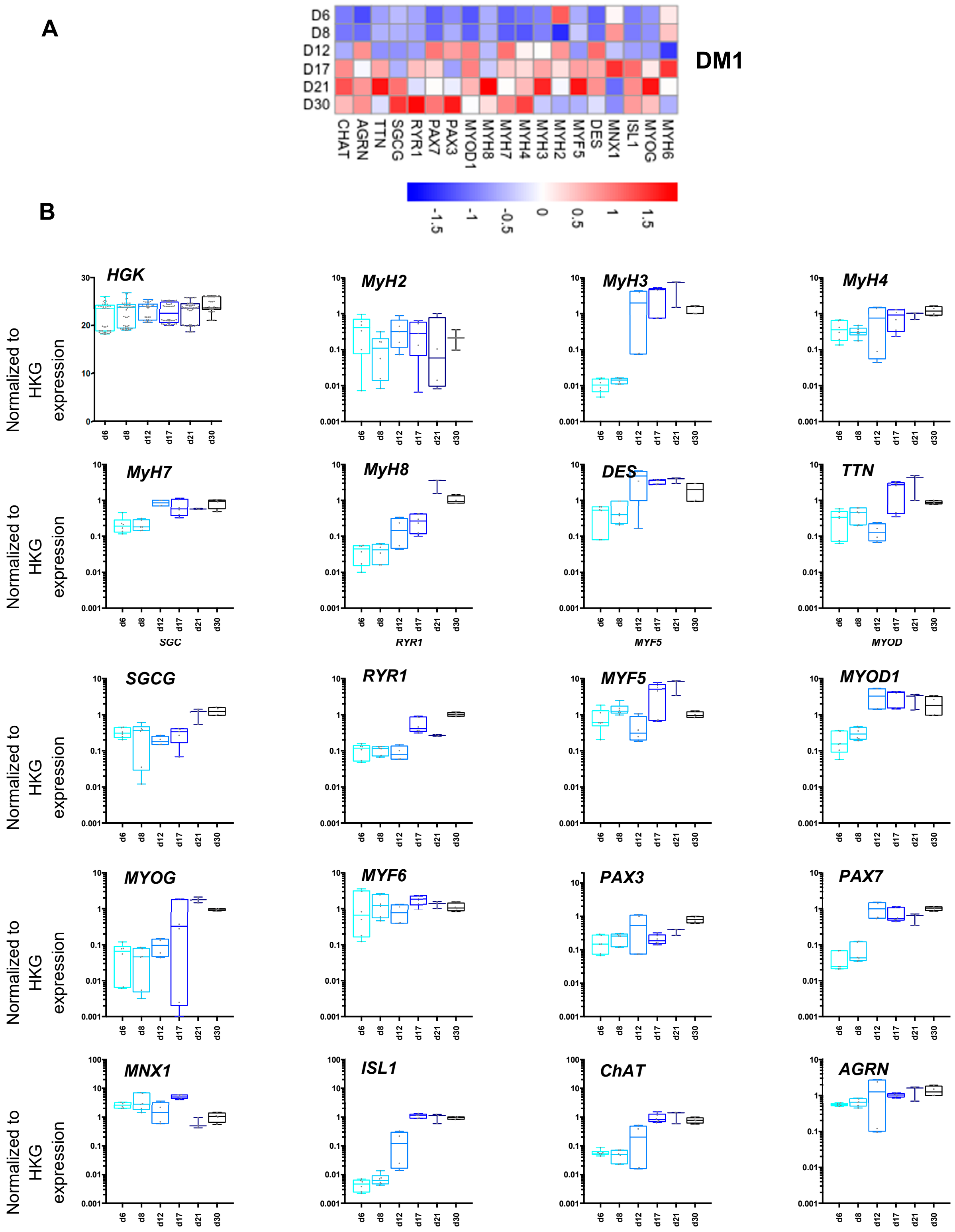

Figure S5. Time course expression of myogenic and motor neurons markers in DM1 hiPSC-derived muscles. (**A**) Heatmap for expression of different muscle and neuronal markers over time for control cells at D6, D8, D12, d17, d21 and D30 post differentiation as determined by RT-qPCR for cells derived from the the GM24559 hiPSC clone. The name of the different genes is indicated below. Expression of the different genes was normalized to the expression level of three different housekeeping genes (*GAPDH*, *HPRT1* and *PPIA)* and to the expression of the different genes at day 30 (ΔΔCT method, n = 6; biological triplicates in technical duplicates). Means ± SEM and statistical significance are reported; Kruskal Wallis multiple comparison test, α = 0.05). (**B**) Expression of different Myosin Heavy Chain genes (*MyH2*, *MyH3*, *MyH4*, *MyH8*) at the different time points and expression of sarcolemmal

proteins such as Desmin (*DES),* a muscle specific class III intermediate filament responsible for connecting myofibrils to each others and to the plasma membrane, *SCGG* encoding a sarcolemmal transmembrane glycoprotein that interacts with Dystrophin, *TITIN* (*TTN*) and *RYR1* (Ryanodine Receptor 1), responsible for Calcium release in the sarcoplasmic reticulum and connection to the transverse tubules, expression of the different transcription factors required for skeletal muscle commitment and differentiation (*MRF4*, *MGN*, *MYF5*, *MYOD1*). Expression of the Paired box protein genes, *PAX3* and *PAX7*. Expression of MNX1 (HB9 homeobox gene 9), *ISLET1* (ISL LIM homeobox 1) motor neurons markers, Cholin-O-Acetyltransferase gene (*ChAT*) expressed by cholinergic neurons and AGRIN required for formation of neuromuscular junction (*AGRN).* Compared to controls, expression of *SGCG* is detectable earlier in DM1. Expression of the *MYF5* and *MYOD1 and MYF6* myogenic marker is activated earlier and remains stable over time while expression of *MYOG* is more variable. *PAX3* is detectable throughout the whole different process with an increase in DM1 cells suggesting maintenance of neuronal/muscle progenitors.

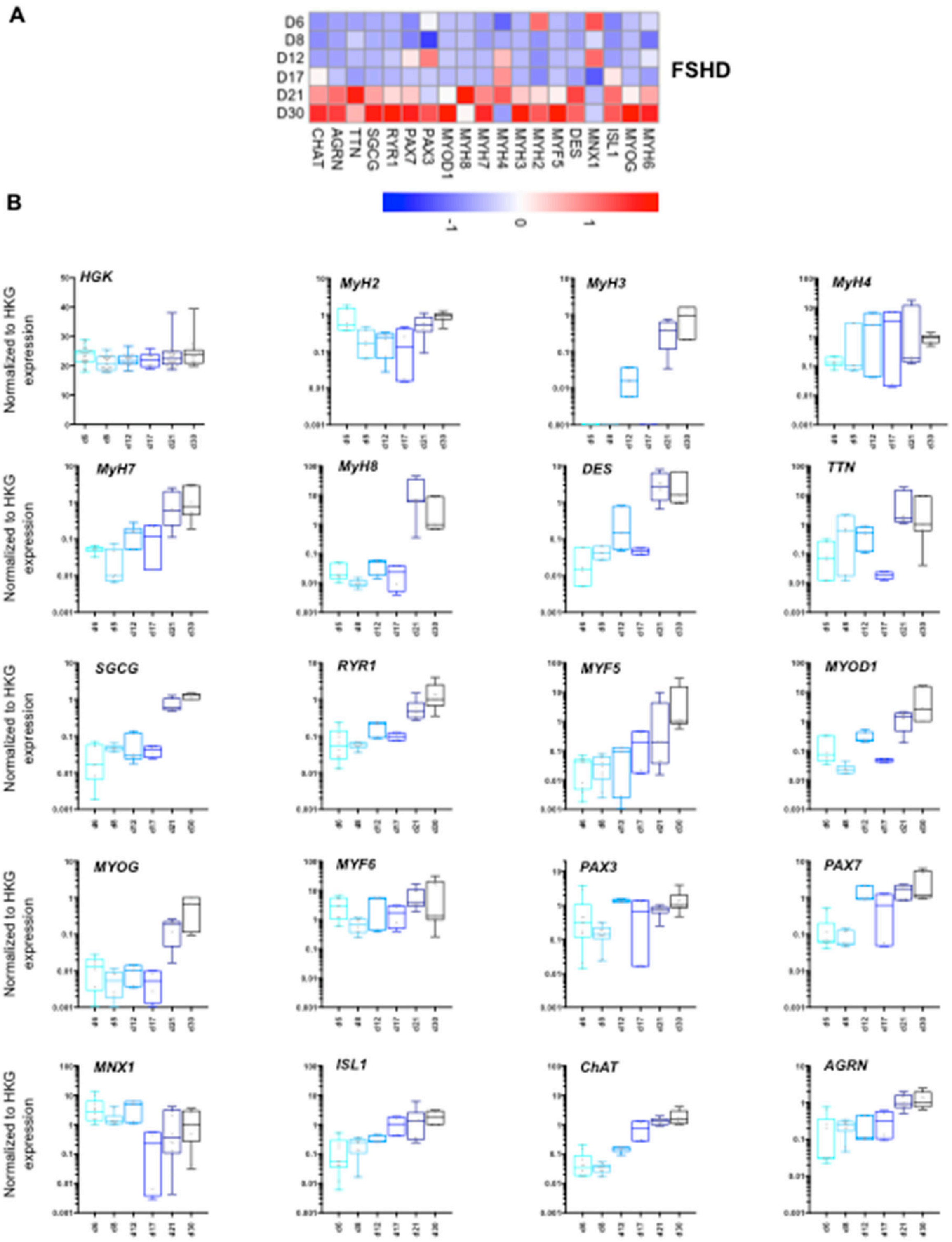

Figure S6. Time course expression of myogenic and motor neurons markers in FSHD hiPSC-derived muscles. (**A**) Heatmap for expression of different muscle and neuronal markers over time for control cells at D6, D8, D12, D17, D21 and D30 post differentiation as determined by RT-qPCR for cells derived from the 14,586 hiPSC clone. The name of the different genes is indicated below. Expression of the different genes was normalized to the expression level of three different housekeeping genes (*GAPDH*, *HPRT* and *PPIA)* and to the expression of the different genes at day 30 (ΔΔCT method, n = 6; biological triplicates in technical duplicates). Means ± SEM and statistical significance are reported; Kruskal Wallis multiple comparison test, $\alpha = 0.05$). (**B**) Expression of different Myosin Heavy Chain genes (*MyH2*, *MyH3*, *MyH4*, *MyH8*) at the different time points and expression of sarcolemmal

proteins such as Desmin (*DES*), a muscle specific class III intermediate filament responsible for connecting myofibrils to each others and to the plasma membrane, *SCGG* encoding a sarcolemmal transmembrane glycoprotein that interacts with Dystrophin, *TITIN* (*TTN*) and *RYR1* (Ryanodine Receptor 1), responsible for Calcium release in the sarcoplasmic reticulum and connection to the transverse tubules, Expression of the different transcription factors required for skeletal muscle commitment and differentiation (*MRF4*, *MGN*, *MYF5*, *MYOD1*). Expression of the Paired box protein genes, *PAX3* and *PAX7*. Expression of MNX1 (HB9 homeobox gene 9), *ISLET1* (ISL LIM homeobox 1) motor neurons markers, Cholin-O-Acetyltransferase gene (*ChAT*) expressed by cholinergic neurons

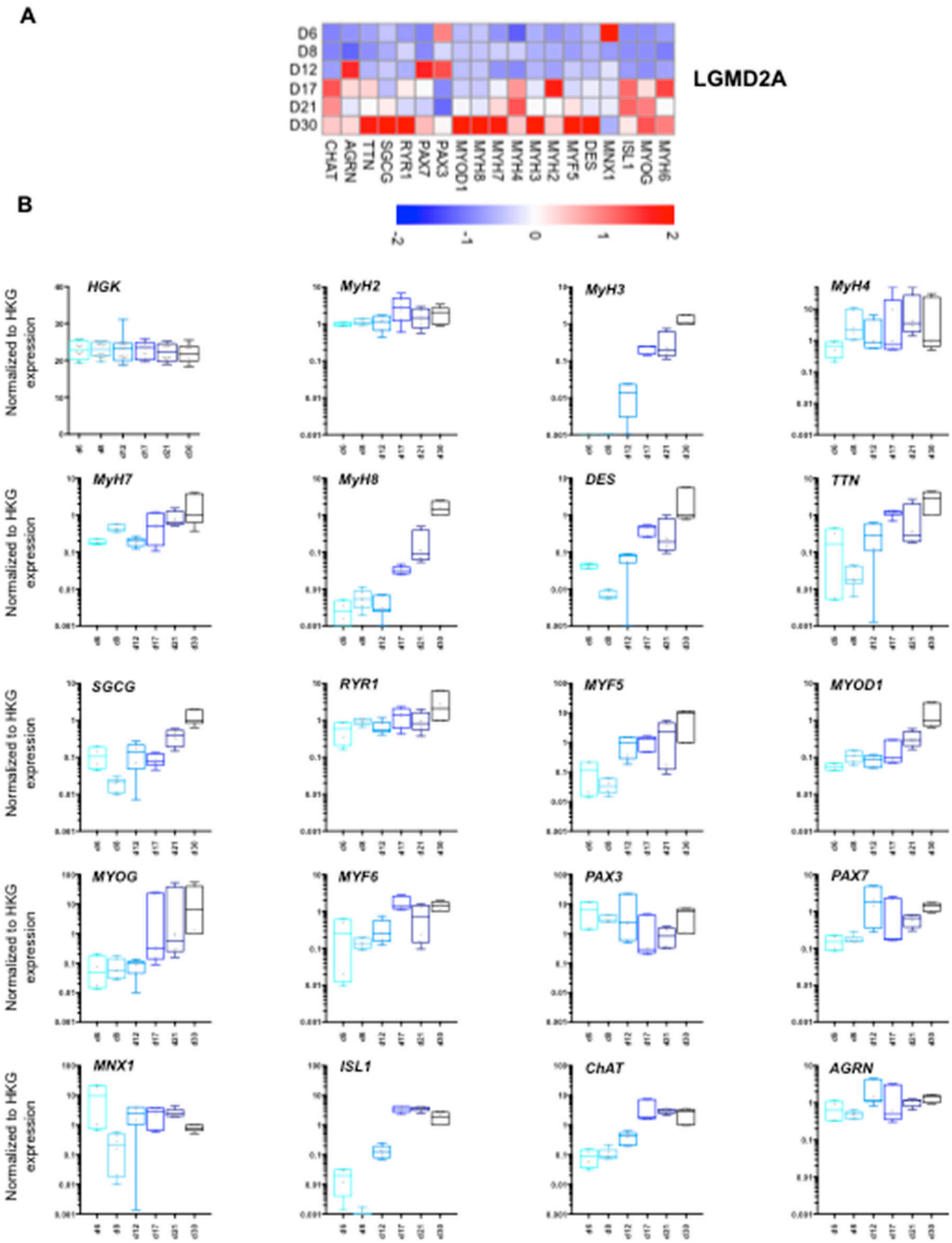

and AGRIN required for formation of neuromuscular junction (*AGRN*). Expression of *MYF6* (MRF4),

is detectable earlier in FSHD. *MNX1* (HB9) expression decreases earlier in FSHD cells while expression of *ISL1* is more progressive.

Figure S7. Time course expression of myogenic and motor neurons markers in LGMD2A hiPSC-derived muscles. (**A**) Heatmap for expression of different muscle and neuronal markers over time for control cells at D6, D8, D12, d17, d21 and D30 post differentiation as determined by RT-qPCR for cells derived from the LMD932C hiPSC clone. The name of the different genes is indicated below. Expression of the different genes was normalized to the expression level of three different housekeeping genes (*GAPDH*, *HPRT1* and *PPIA)* and to the expression of the different genes at day 30 (ΔΔCT method, n = 6; biological triplicates in technical duplicates). Means ± SEM and statistical significance are reported; Kruskal Wallis multiple comparison test, α = 0.05). (**B**) Expression of different Myosin Heavy Chain genes (*MyH2*, *MyH3*, *MyH4*, *MyH8*) at the different time points and expression of sarcolemmal proteins such as Desmin (*DES),* a muscle specific class III intermediate filament responsible for connecting myofibrils to each others and to the plasma membrane*, SCGG* encoding a sarcolemmal transmembrane glycoprotein that interacts with Dystrophin*, TITIN* (*TTN*) and *RYR1* (Ryanodine Receptor 1), responsible for Calcium release in the sarcoplasmic reticulum and connection to the transverse tubules, Expression of the different transcription factors required for skeletal muscle commitment and differentiation (*MRF4*, *MGN*, *MYF5*, *MYOD1*). Expression of the Paired box protein genes, *PAX3* and *PAX7*. Expression of MNX1 (HB9 homeobox gene 9), *ISLET1* (ISL LIM homeobox 1) motor neurons markers, Cholin-O-Acetyltransferase gene (*ChAT*) expressed by cholinergic neurons and AGRIN required for formation of neuromuscular junction (*AGRN).* We observed an early activation of *RYR1* in LGMD2A compared to controls. *MNX1* (HB9) expression is very variable in LGMD2A while expression of *ISL1* is more progressive.

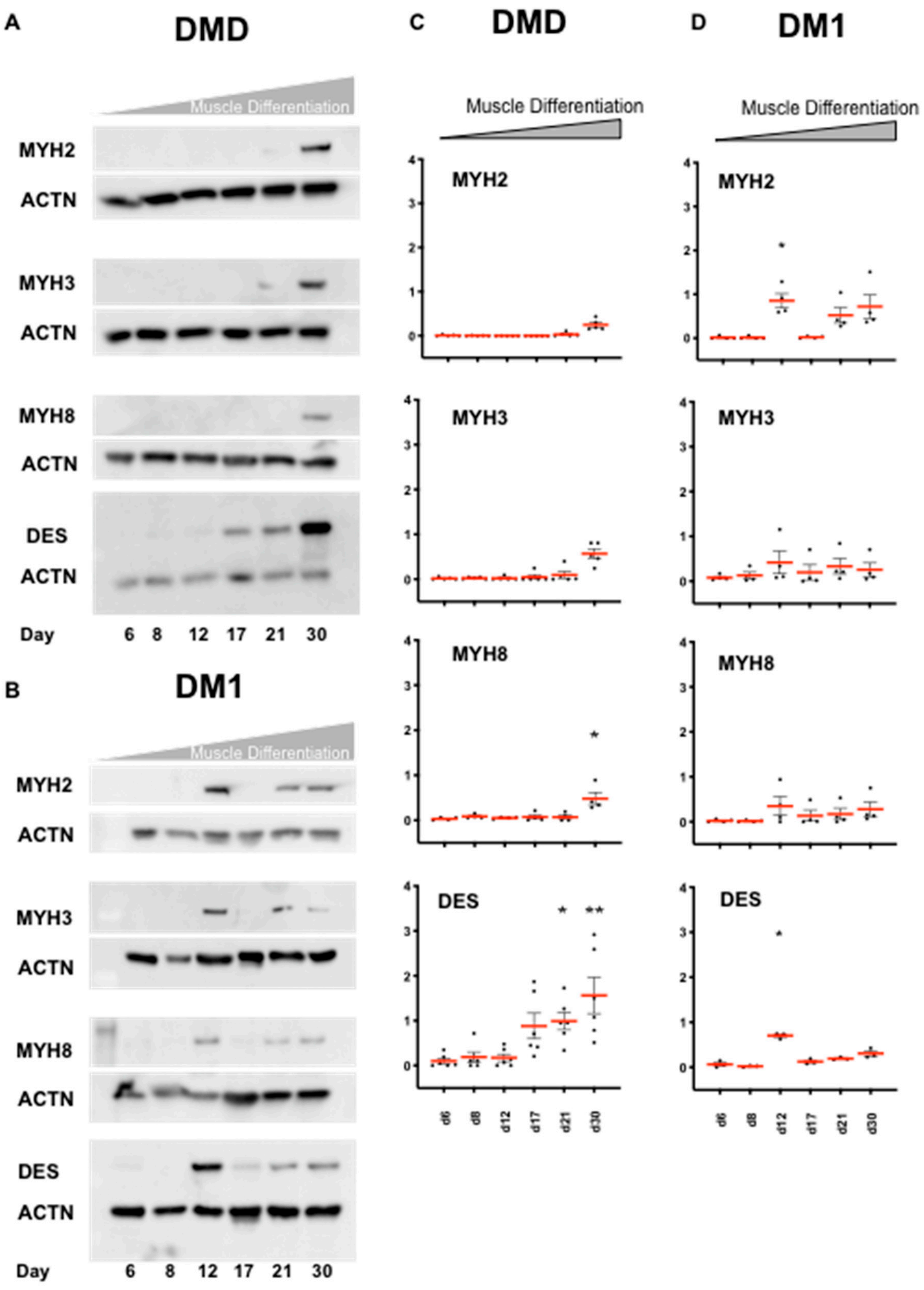

Figure S8. Quantification of myogenic markers protein levels in DMD and DM1 hiPSC-derived muscles. (**A**,**B**) Representative Western blot on whole cell extracts performed at different time points post differentiation, D6, 8, 12, 17, 21, 30 using antibodies against MyH2, MyH3, MyH8 or Desmin. Beta Actin (ACTN) was used as loading control for DMD (**A**) and DM1 (**B**). (**D**,**C)** Quantification of ratio between each muscle marker and Beta Actin at the different time points. Quantification corresponds to the average of three independent experiments for DMD (**C**) and DM1 (**D**). Means ±

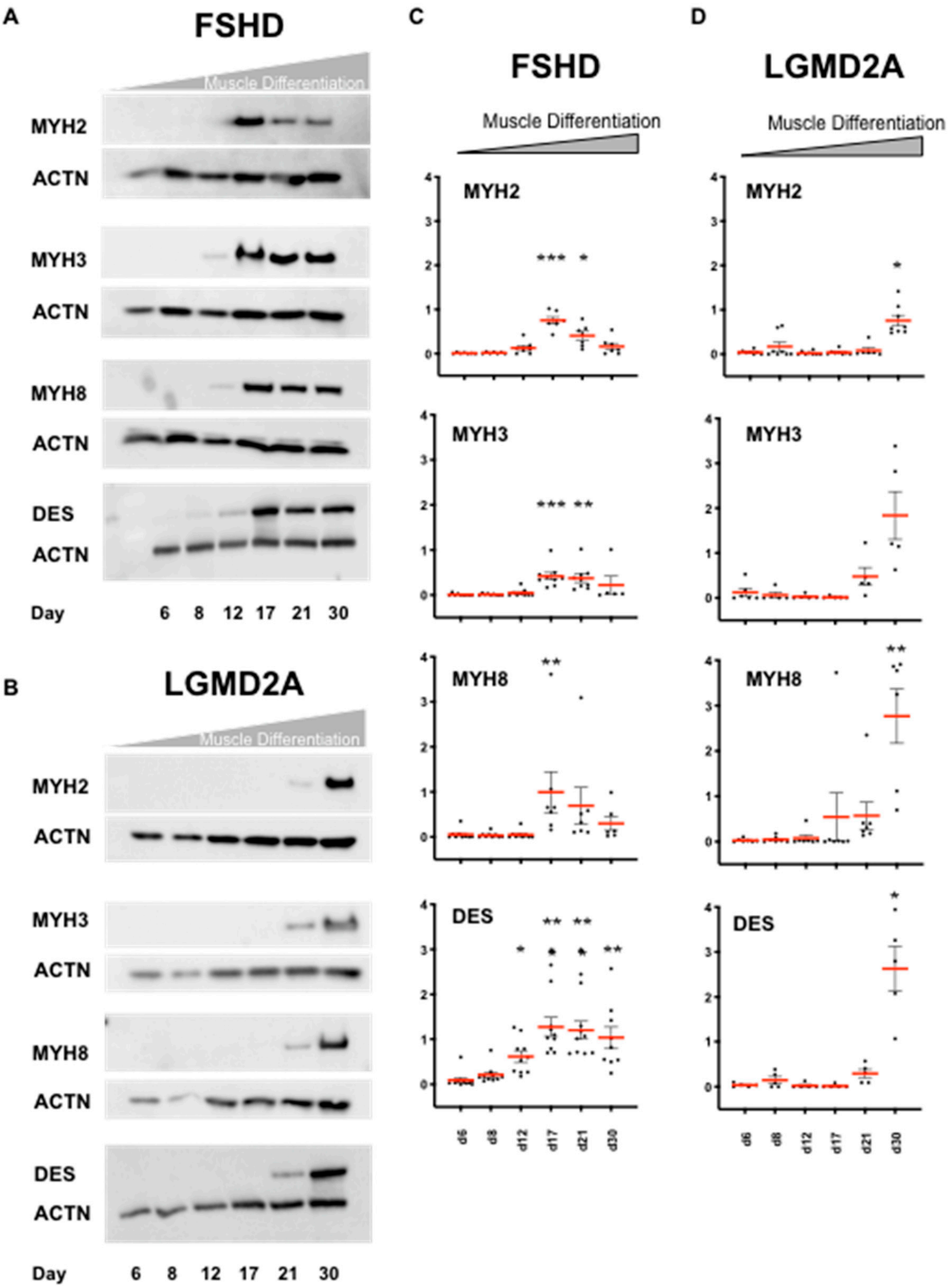

SEM and statistical significance are reported; Kruskal Wallis multiple comparison test, α = 0.05). * p <0.05; ** $p < 0.01$; *** $p < 0.005$; **** $p < 0.001$.

Figure S9. Quantification of myogenic markers protein levels in FSHD and LGMD2A hiPSC-derived muscles. (**A**,**B**) Representative Western blot on whole cell extracts performed at different time points post differentiation, D6, 8, 12, 17, 21, 30 using antibodies against MyH2, MyH3, MyH8 or Desmin. Beta Actin (ACTN) was used as loading control in FSHD (**A**) and LGMD2A (**B**). (**C**,**D)** Quantification

of ratio between each muscle marker and Beta Actin at the different time points in FSHD (**C**) and LGMD2A (**D**). Quantification corresponds to the average of three independent experiments. Means ± SEM and statistical significance are reported; Kruskal Wallis multiple comparison test, $\alpha = 0.05$). *p <0.05; ** $p < 0.01$; *** $p < 0.005$; **** $p < 0.001$.

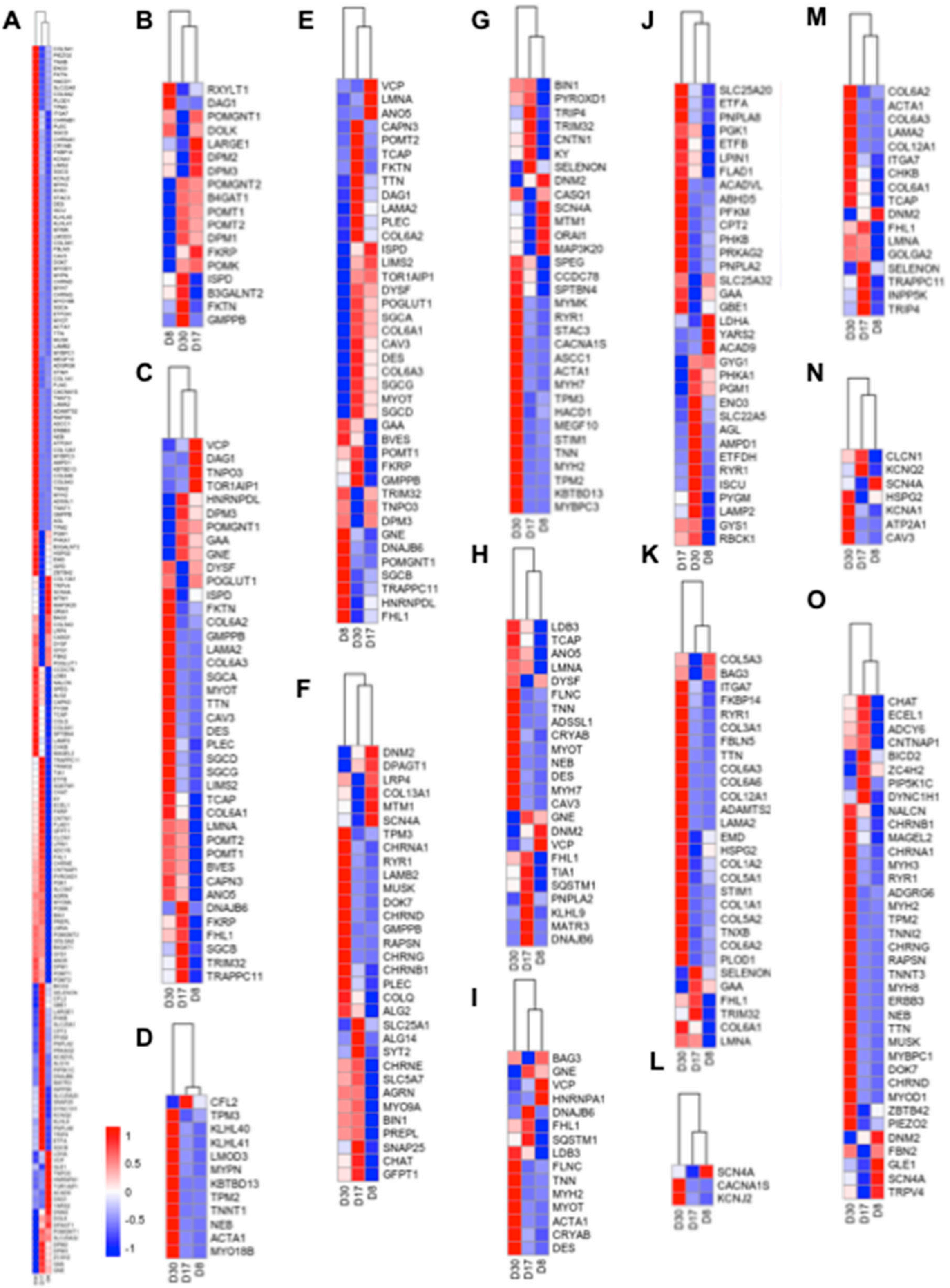

Figure S10. Heatmap of RNAseq data for genes involved in muscular dystrophies. (**A**) We selected genes used in whole exome sequencing panels for diagnostics of muscle diseases and compared expression of 193 genes at the different time points (D8, D17, D30) (TPM values, distance: Manhattan, Clustering: Ward.D2) in muscle cells derived from control hiPSCs. The muscle whole exome sequencing panels used as a reference contains 194 genes but the *PGAM2* is not expressed. For all heat maps the order was determined by clustrering and dendrograms branches and not by the time course expression. (**B–N**) List of genes for (**B**) Alpha dystroglycanopathies. (**C**) Scapular girdle

dystrophies. (**D**) Congenital myopathies; Nemaline myopathies. (**E**) Limb girdle myopathies. (**F**) Congenital myasyteny. **G**. Congenital myopathies, non-Nemaline. (**H**) Scapuloperoneal myopathy. (**I**) Myofibrillar myopathies. (**J**) Metabolic myopathies. (**K**) Congenital myopathy with retraction. (**L**) Periodical paralysis. (**M**) Muscular dystrophies, non dystroglycanoapthies. (**N**) Non dystrophic myopathies. (**O**) Arthrogryposis.

Supplemental movie 1. Recoding of myofibers after 30 days post differentiaton

Supplemental movie 2. Recording of Calcium transients after addition to the cell culture medium of FLUO-8AM fluorescent Ca^{2+} indicator recorded using a Fast Imaging Observer system (Axio Observer.Z1/7; Zeiss) with a 10X lens. Fluorescence was excited at 488nm and emission collected at >509 nm. Small green dots corresponds to neuron while muscle fibers are clearly visible in the center of the screen.

References

1. Badja, C.; Maleeva, G.; El-Yazidi, C.; Barruet, E.; Lasserre, M.; Tropel, P.; Binetruy, B.; Bregestovski, P.; Magdinier, F. Efficient and cost-effective generation of mature neurons from human induced pluripotent stem cells. *STEM CELLS Transl. Med.* **2014**, *3*, 1467–1472, doi:10.5966/sctm.2014-0024.
2. Dion, C.; Roche, S.; Laberthonnière, C.; Broucqsault, N.; Mariot, V.; Xue, S.; Gurzau, A.D.; Nowak, A.; Gordon, C.T.; Gaillard, M.-C.; et al. SMCHD1 is involved inde novomethylation of theDUX4-encoding D4Z4 macrosatellite. *Nucleic Acids Res.* **2019**, *47*, 2822–2839, doi:10.1093/nar/gkz005.
3. Bolger, A.M.; Lohse, M.; Usadel, B. Trimmomatic: a flexible trimmer for Illumina sequence data. *Bioinformatics* **2014**, *30*, 2114–2120, doi:10.1093/bioinformatics/btu170.
4. Dobin, A.; Davis, C.A.; Schlesinger, F.; Drenkow, J.; Zaleski, C.; Jha, S.; Batut, P.; Chaisson, M.; Gingeras, T.R. STAR: ultrafast universal RNA-seq aligner. *Bioinform.* **2012**, *29*, 15–21, doi:10.1093/bioinformatics/bts635.
5. Yu, G.; Wang, L.-G.; Han, Y.; He, Q.-Y. clusterProfiler: an R Package for Comparing Biological Themes Among Gene Clusters. *OMICS: A J. Integr. Boil.* **2012**, *16*, 284–287, doi:10.1089/omi.2011.0118.
6. Broucqsault, N.; Morere, J.; Gaillard, M.-C.; Dumonceaux, J.; Torrents, J.; Salort-Campana, E.; De Paula, A.M.; Bartoli, M.; Fernandez, C.; Chesnais, A.L.; et al. Dysregulation of 4q35- and muscle-specific genes in fetuses with a short D4Z4 array linked to facio-scapulo-humeral dystrophy. *Hum. Mol. Genet.* **2013**, *22*, 4206–4214, doi:10.1093/hmg/ddt272.
