# Supplementary figures and images for: Multilineage Differentiation for Formation of Innervated Skeletal Muscle Fibers from Healthy and Diseased Human Pluripotent Stem Cells
Source: Cells. 2020 Jun 23;9(6):1531. doi: 10.3390/cells9061531 (PMC7349825; doi:10.3390/cells9061531)

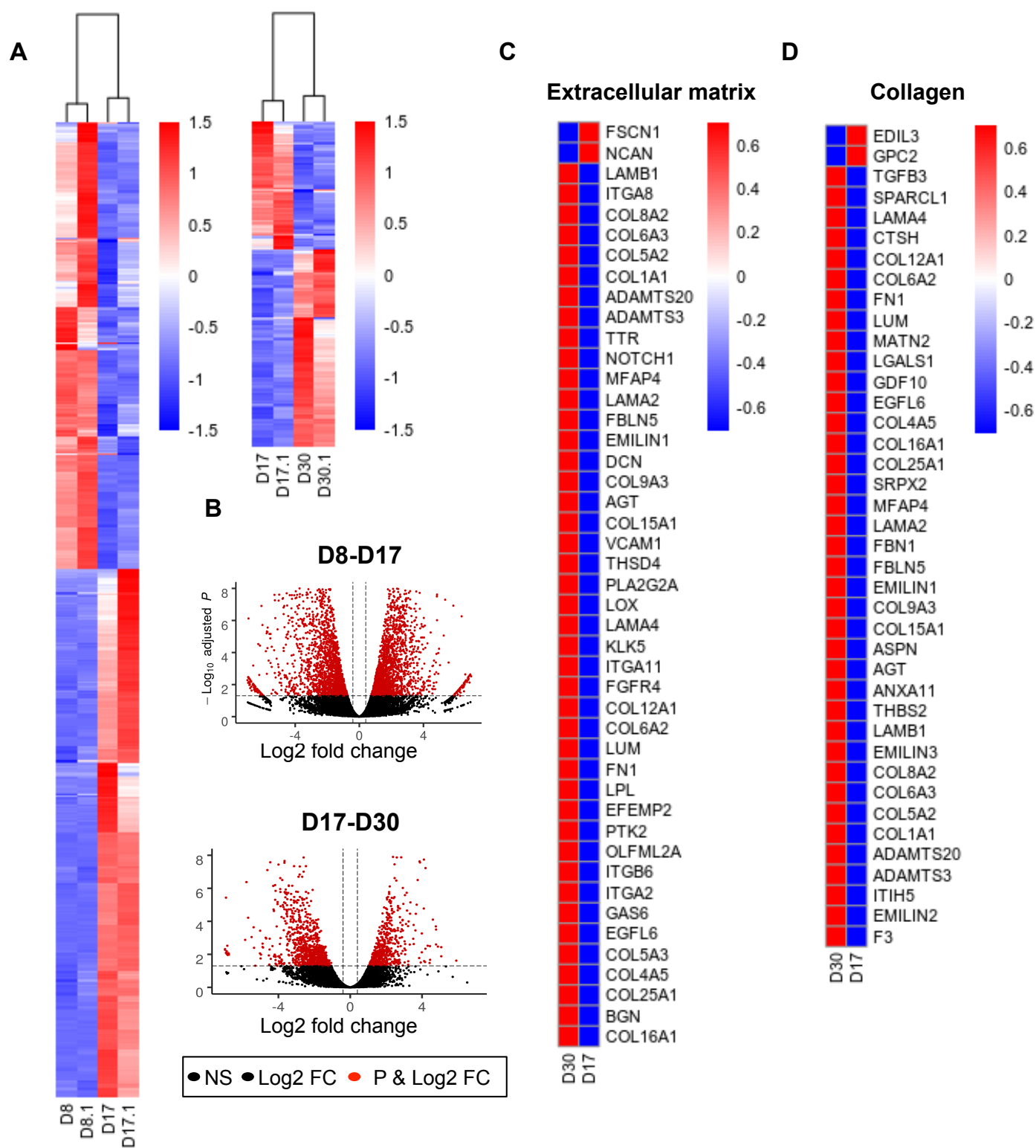

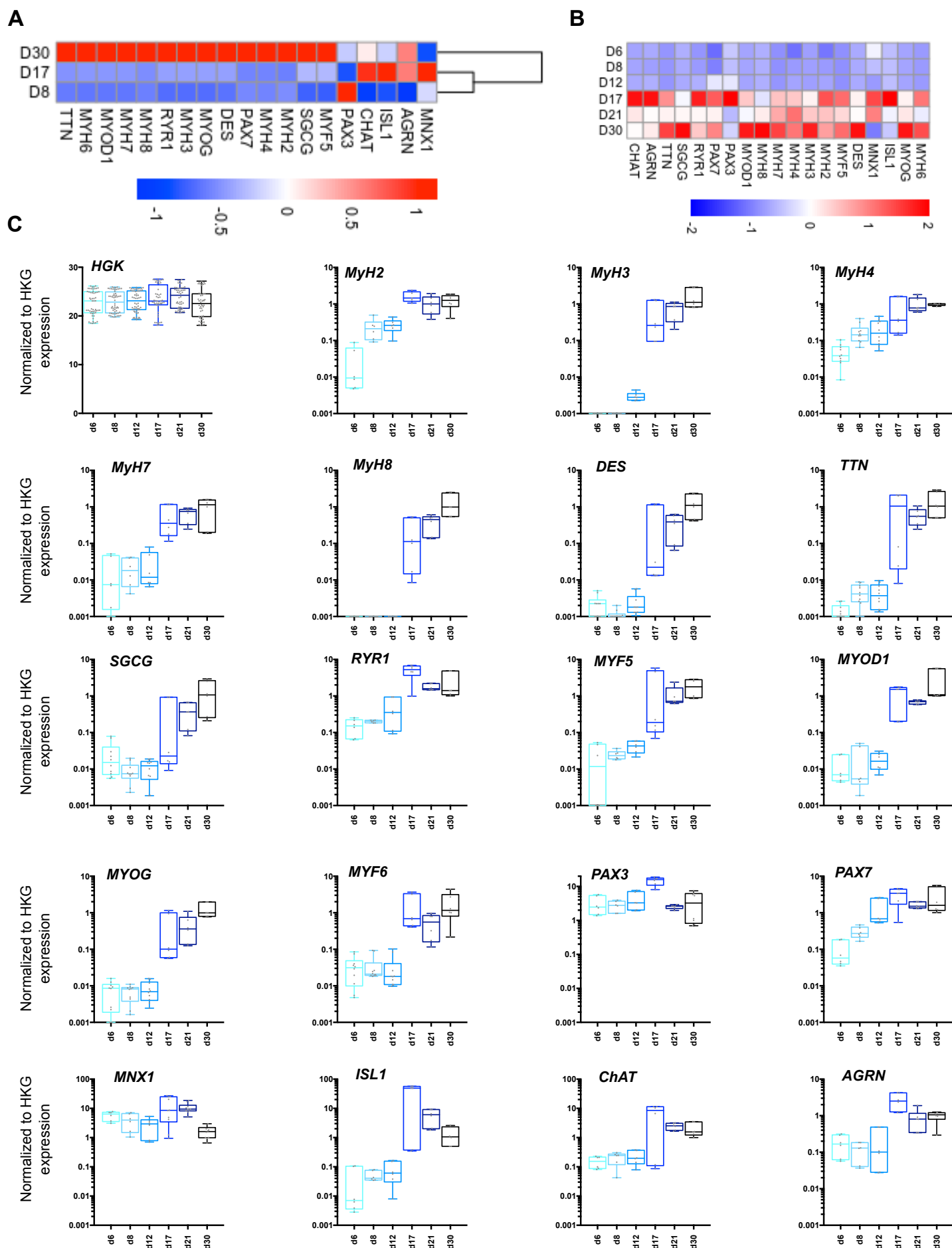

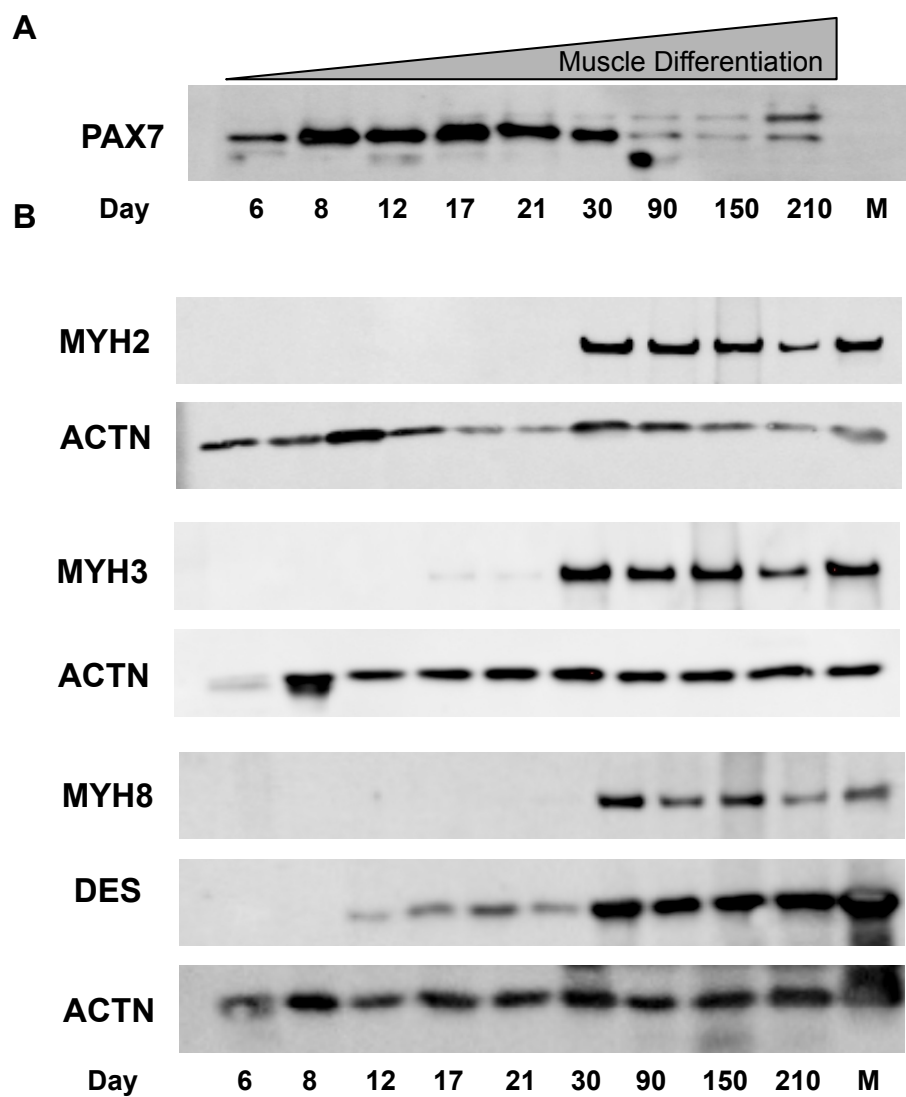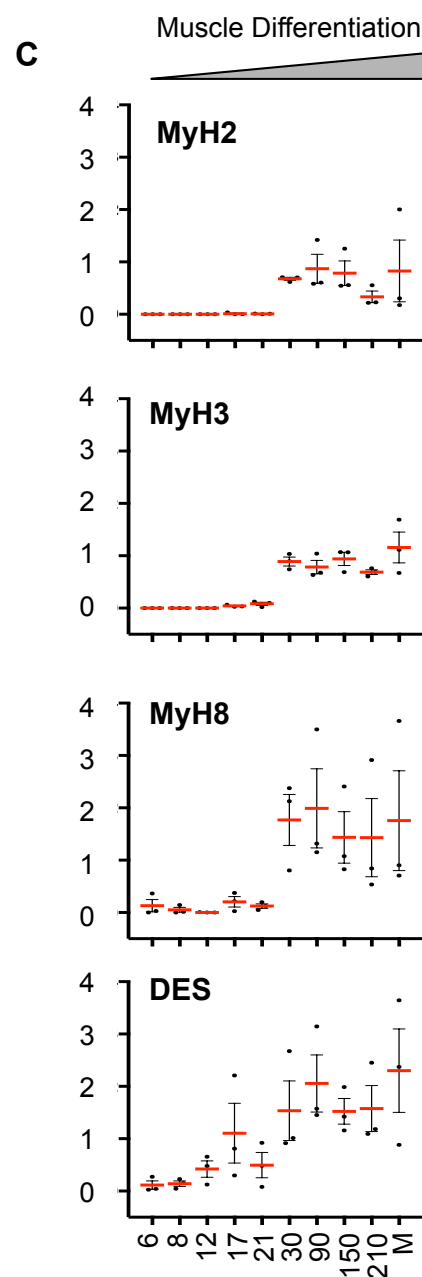

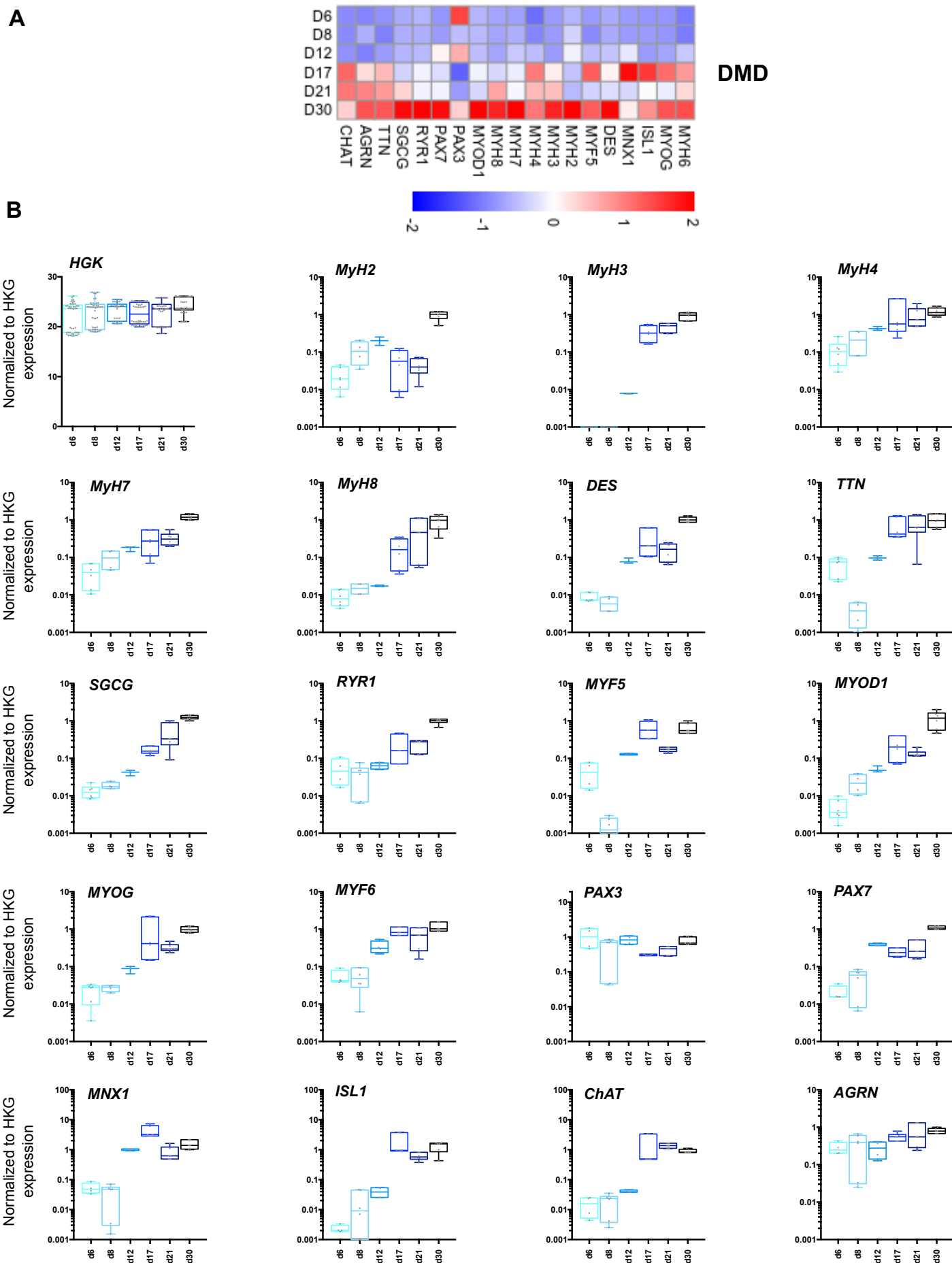

**A**

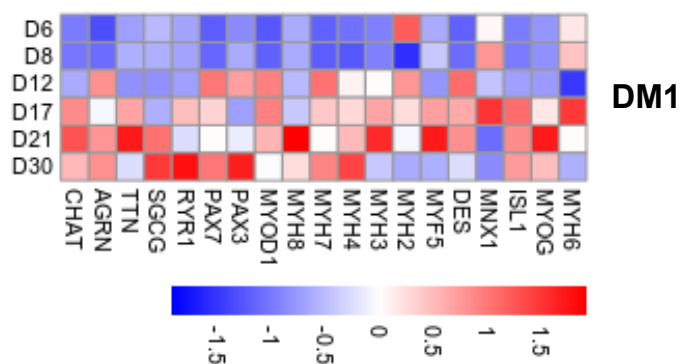

**B**

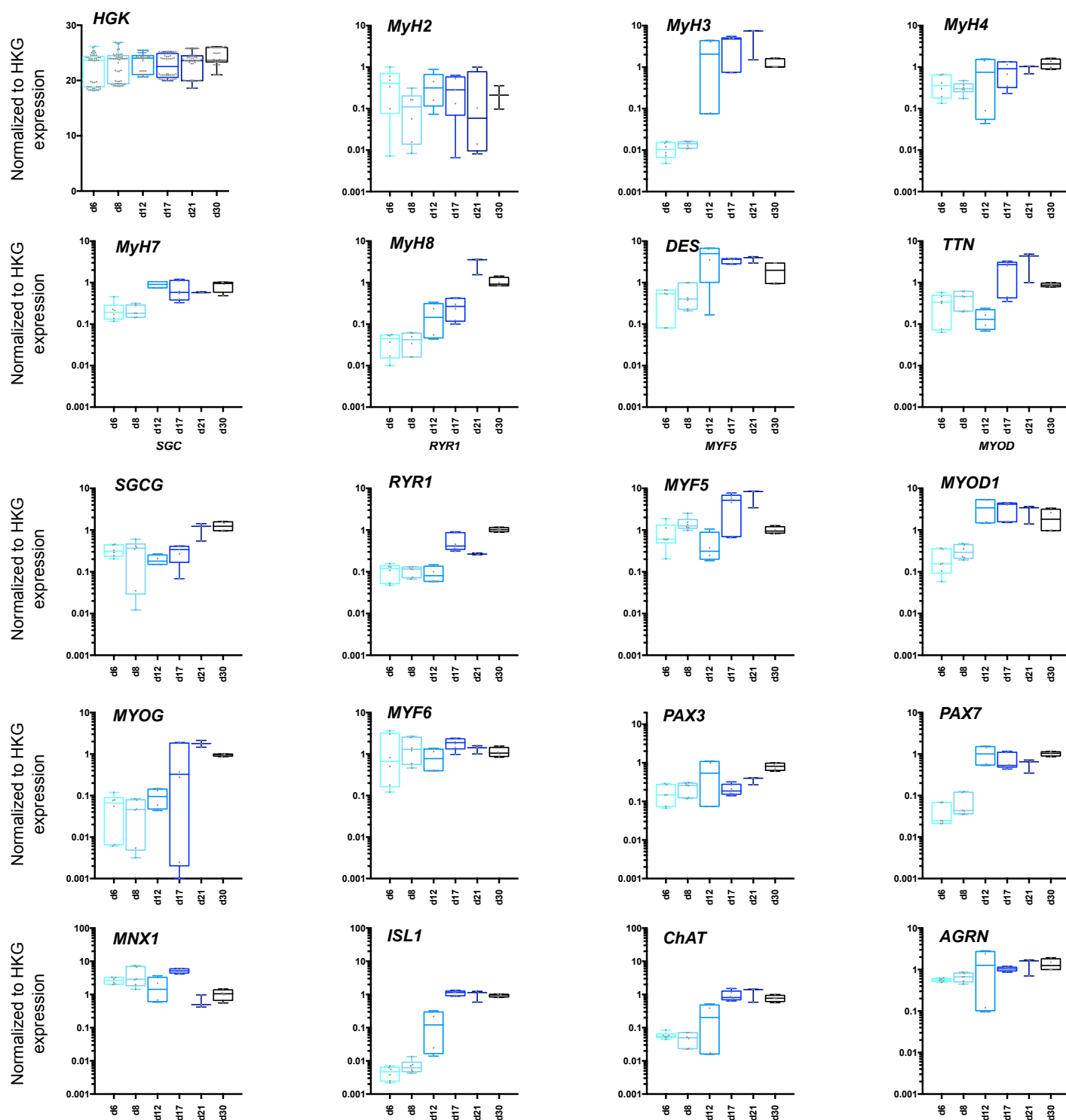

**A**

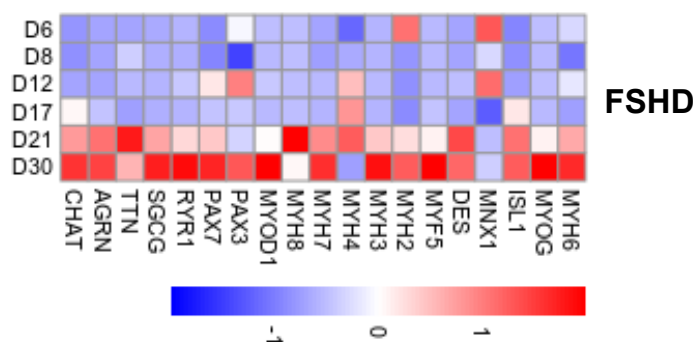

**B**

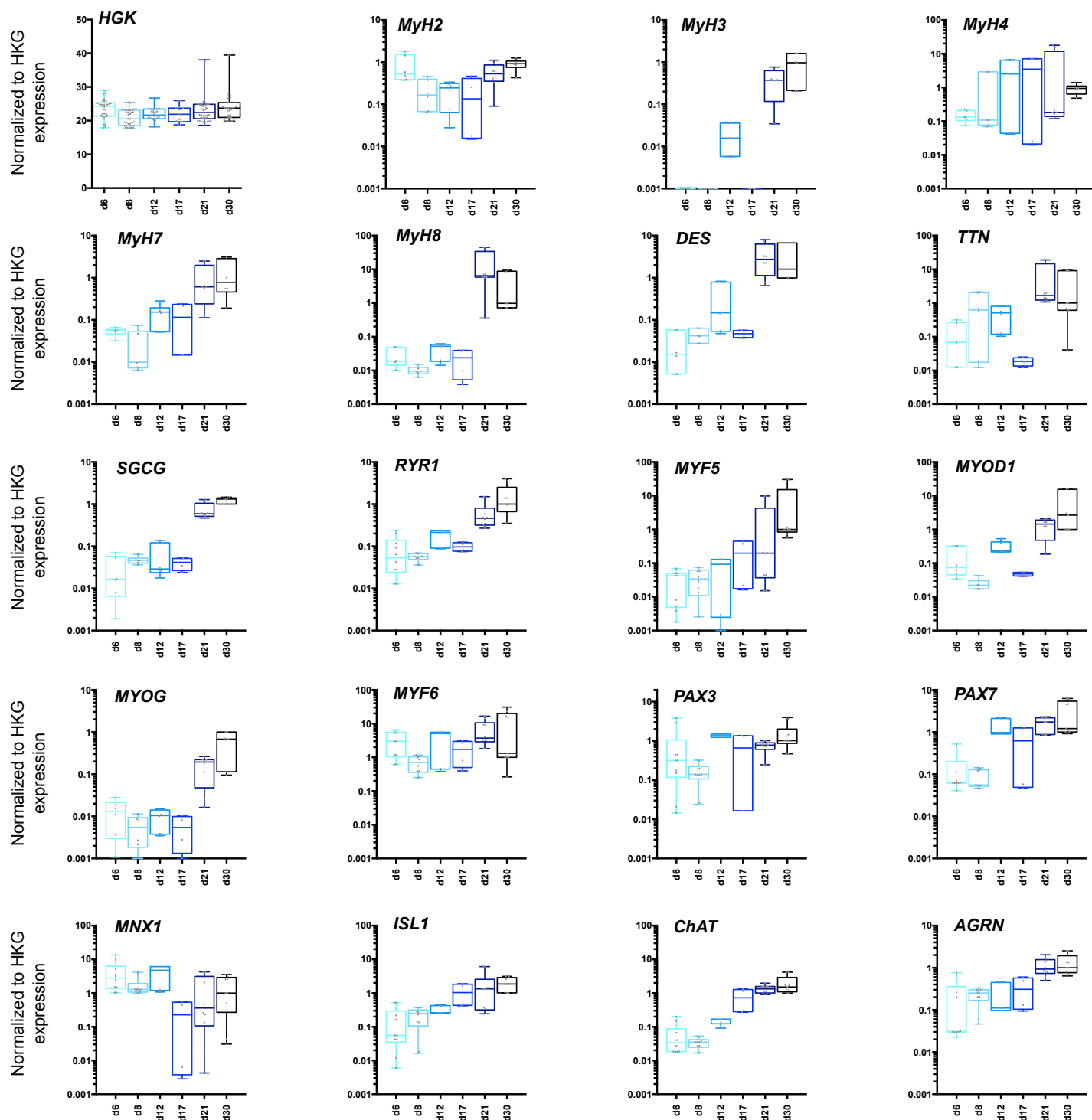

**A**

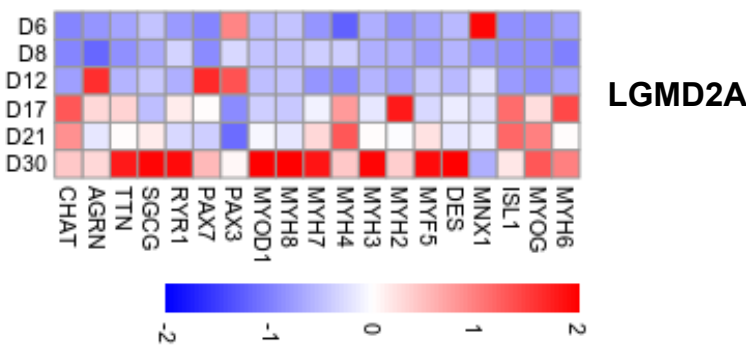

**B**

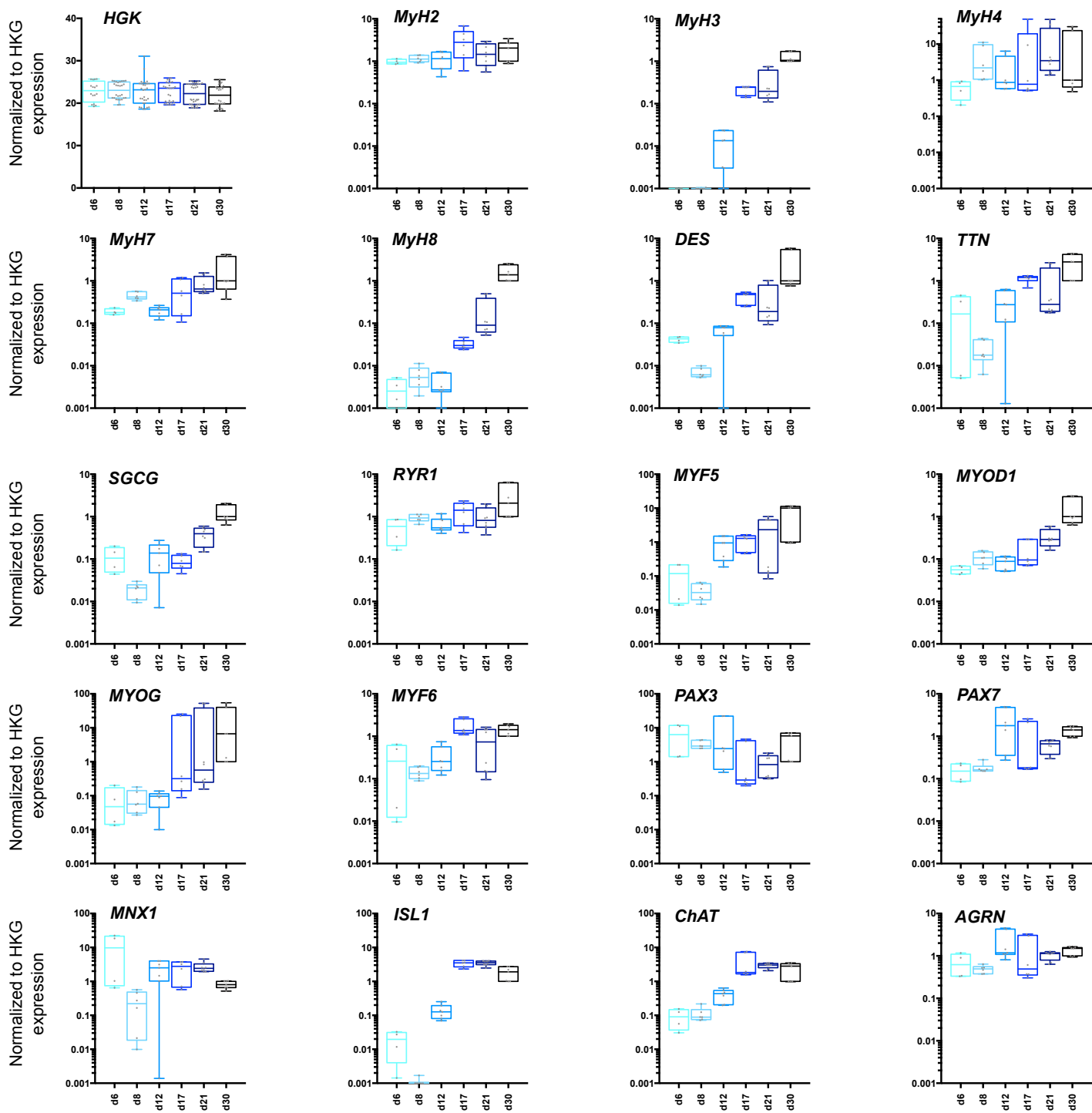

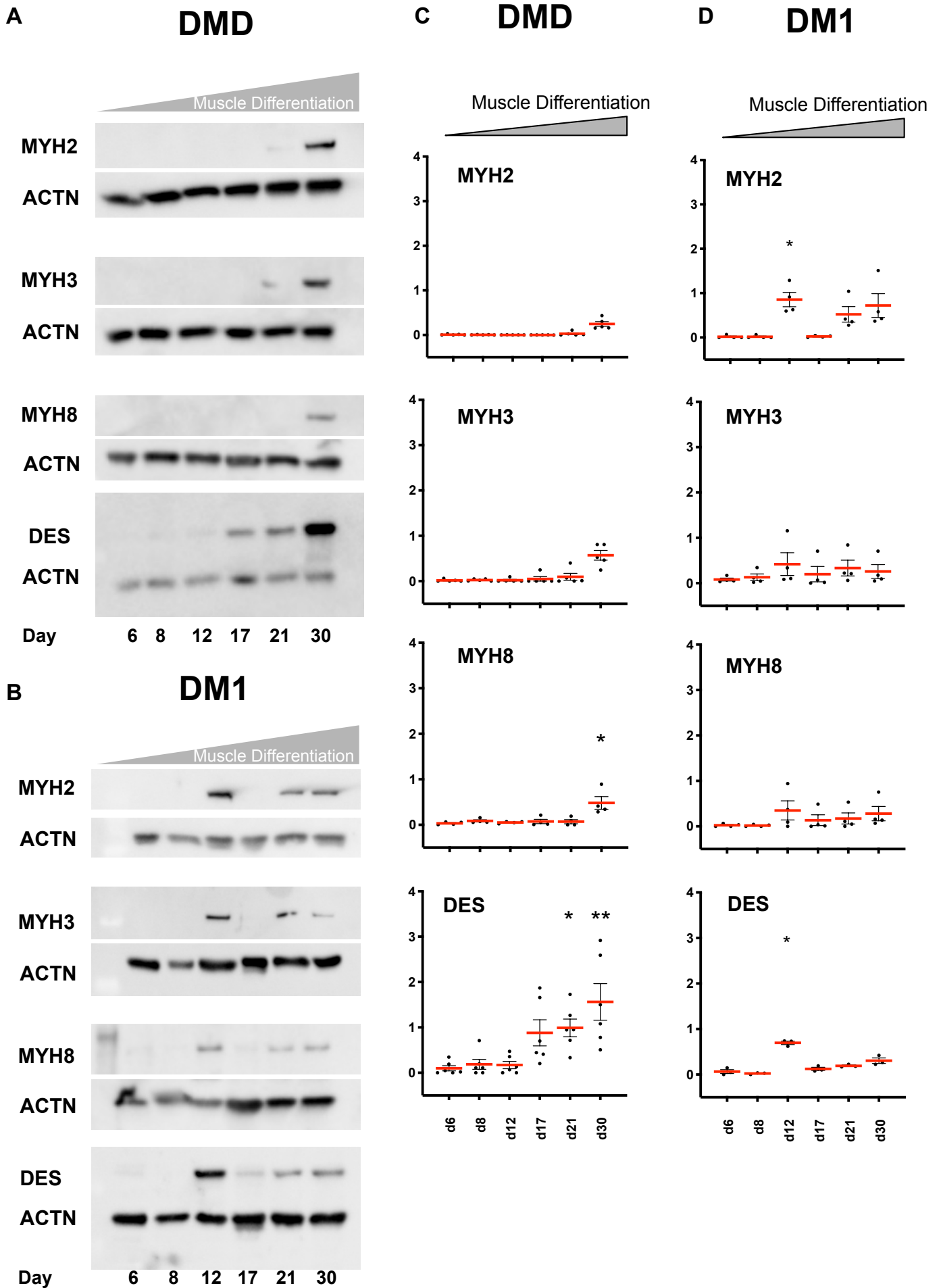

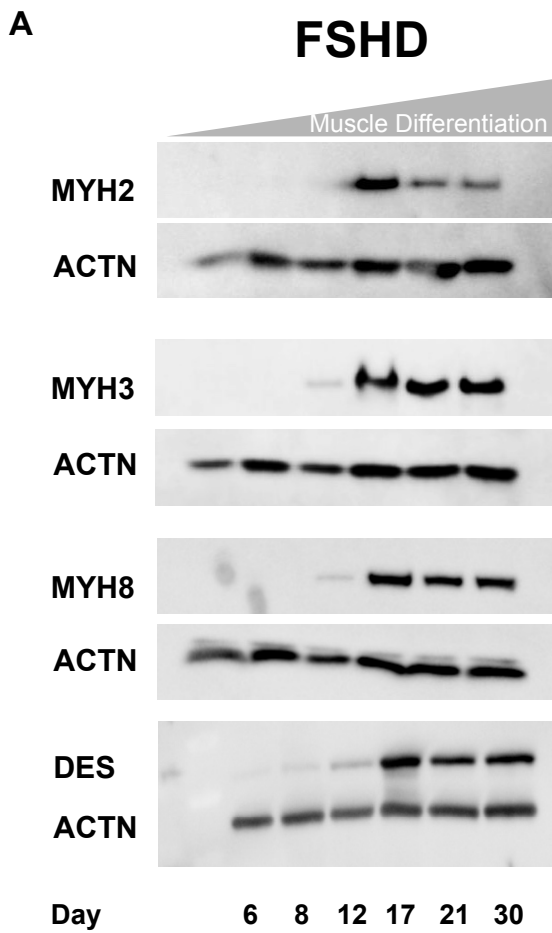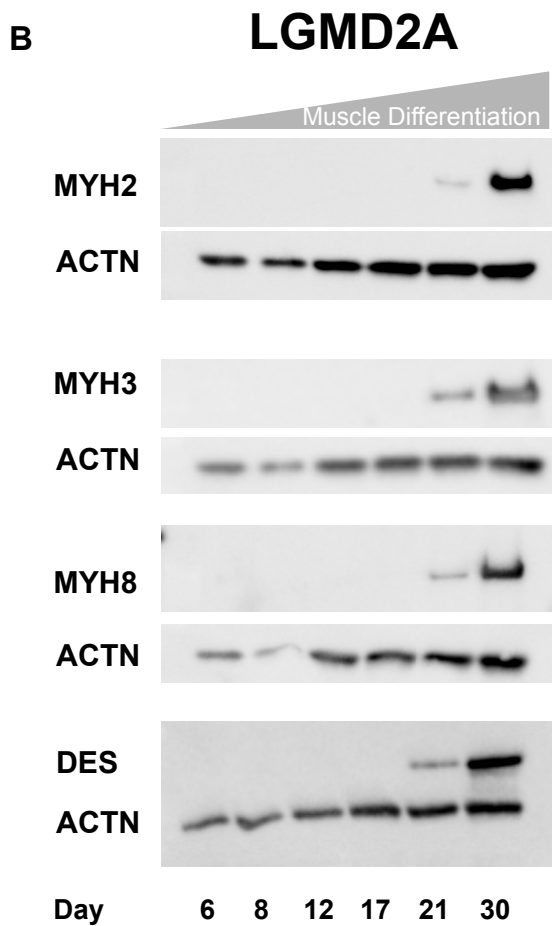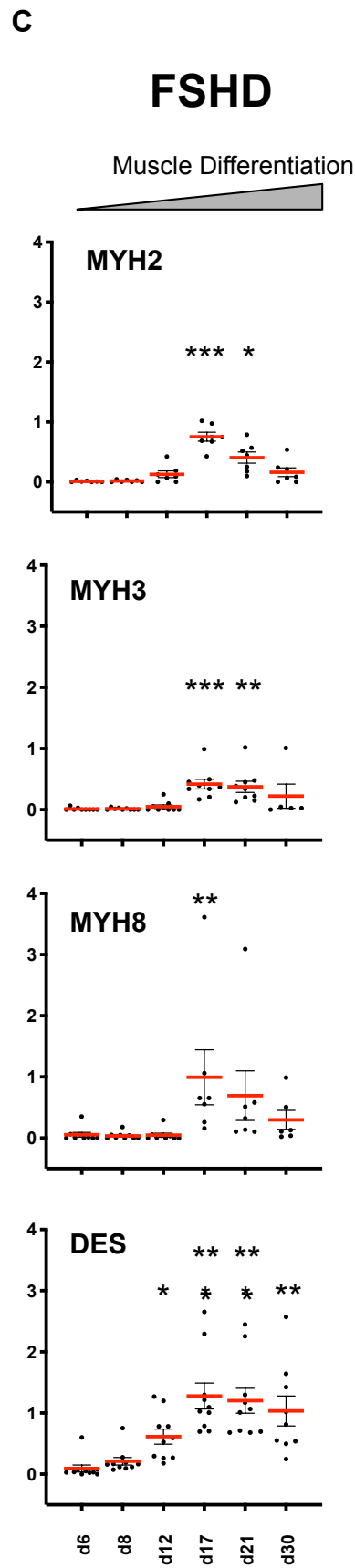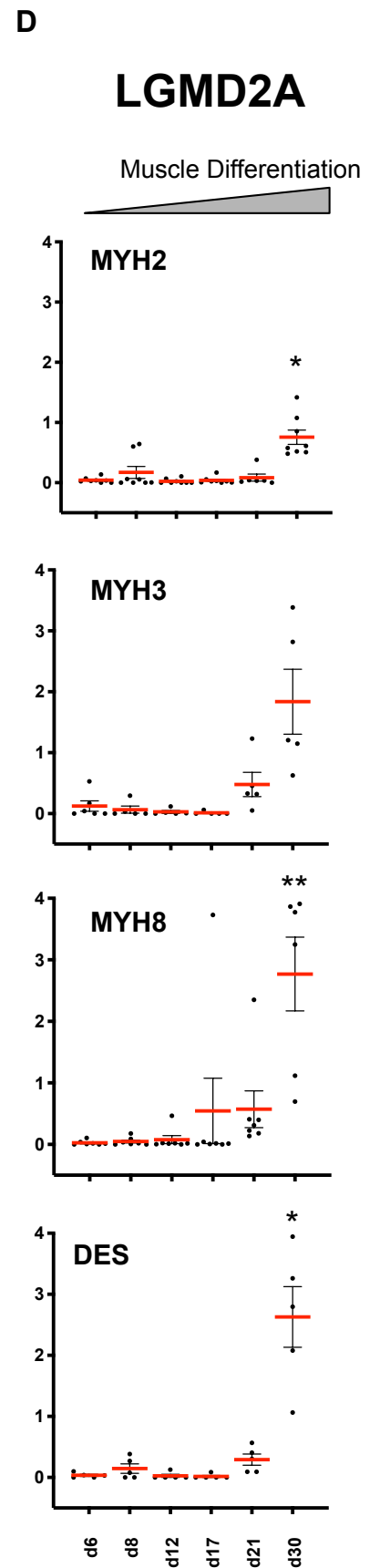

Supplement: Supplementary file 1 [file cells-09-01531-s001.zip › Mazaleyrat et al. Supplementary figures 1-10.pdf]
